# Thioglycoside functionalization *via* chemoselective phosphine acceleration of a photocatalytic thiol–ene reaction†

**DOI:** 10.1039/d5cc00131e

**Published:** 2025-04-29

**Authors:** Arun K. Thangarasu, Charlie Fehl

**Affiliations:** a Chemistry Department, Wayne State University Detroit MI 48202 USA charlie.fehl@wayne.edu

## Abstract

Thioglycosides are enzymatically stable carbohydrate variants used in biotechnology as probes and investigational drugs. To date, harsh activation conditions limit the scope of thiol–ene sugar ligations. Here, we show that phosphines act as a photoredox mediator to accelerate radical thiol–ene reactions between thiosugars and olefins, enabling mild visible light-driven, ambient, and fully aqueous conditions.

## Abstract 2

Green, operationally simple, and chemoselective access to thioglycoside analogs using mild photoredox condition in water.

## Graphical abstract

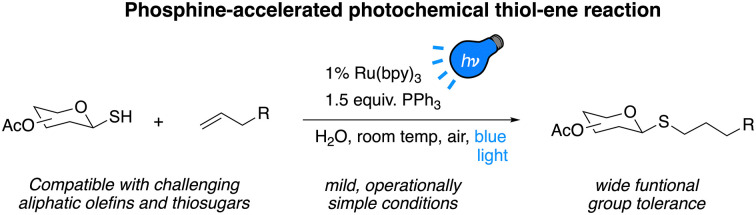

Glycans, one of the major classes of biomolecules, have diverse biological functions that are directly linked to their highly variable molecular structures.^1,2^ These compounds are undeniably important in biology, but their wide structural diversity poses significant challenges for their synthesis. Methods to access sugars are critical to advance biotechnology. To meet this challenge, many reactions for forming glycosidic bonds have been developed, each with unique advantages that enable researchers to tackle these challenges and access a wide variety of substituted glycans. One important sugar class are thioglycosides, which are distinguished by substituting the glycosidic oxygen with sulfur.^3,4^ These compounds can function as glycoside mimics whilst resisting enzymatic cleavage by *O*-glycosidases. Indeed, thioglycosides are used as sugar probes^5^ and are found in clinical trials as the investigational drugs olitigaltin^6^ and glucoraphanin.^7^ Synthetic strategies for glycoside analogs often employ radical reactions because they offer wide functional group compatibility and can preserve the anomeric selectivity of carbohydrate precursors.^8–10^ Radical glycosylation is an increasingly used method for producing carbohydrates.^11^ Typically, *C*-glycosides are formed *via* radical glycosylation,^12–14^ whereas only a few radical methodologies, like thiol–ene reactions, have been used for *S*-glycosides.^15,16^

Photochemical conditions can activate thiyl radicals in thiol–ene reactions. A mild thiol–ene variant involving visible light and a ruthenium bipyrazyl (Rubpz) catalyst is known to activate benzylic thiols and styrenes (Fig. 1a).^17^ However, carbohydrates, more challenging to oxidize than aliphatic thiols, require harsher photocatalytic conditions such as ultraviolet (UV) irradiation and excess of a photoinitator like 2,2-dimethoxy-2-phenylacetophenone (DPAP). DPAP and UV light enable thiol–ene reactions between thiosugars and glycals (Fig. 1b).^18,19^ These reaction yields are significantly enhanced when conducted at lower temperatures to circumvent these harsh activation conditions.^20^ Therefore, access to thiol–ene reactions that couple aliphatic olefins with thiosugars under mild, green conditions still remains a challenge in carbohydrate chemistry.

**Fig. 1:**
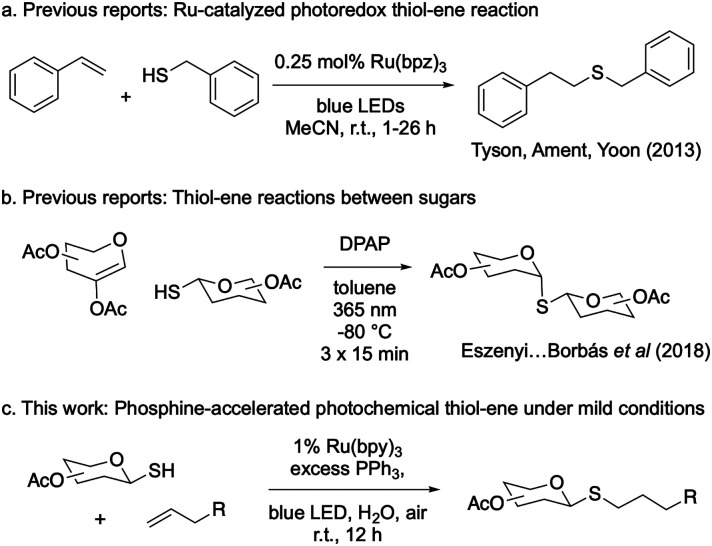
Previous and present work on photocatalytic thiol–ene reactions.

While optimizing thiol–ene chemistry on sugars, we discovered a pathway to facilitate reaction on a variety of olefin substrates in ambient, aqueous conditions using just visible light for the initiation energy (Fig. 1c). By using triphenylphosphine as a reaction “accelerator,” the weak oxidation potential of ruthenium bipyridyl (Rubpy) catalysts afforded a diverse variety of substituted glycosyl thioethers in high yields from glucose and galactose (Fig. 1c). Our findings revealed that triphenylphosphine facilitated *S*-glycoside formation even with substrates known to have low thiol–ene reactivity.^17,21^ This mild, chemoselective thiol–ene glycosylation reaction was easy to perform, as it used ambient, fully aqueous, aerobic conditions and a blue light emitting diode (LED) lightsource.

At the onset of our investigation, recent reports on UV-light desulfurization of thiols^22–24^ inspired us to investigate the use of 1-thiolsugars in a radical pathway for synthesizing *C*-glycosides. We initially sought to develop a method for visible light-triggered photocatalytic *C*-glycosylation that uses phosphine-directed radical desulfurization by utilizing anomeric thiols as donor molecules. The synthesis of 1-thiosugar donors 1–4 was synthesized by employing the protocol developed by Dong *et al.* (Fig. 2A).^25^ Based on existing reports, we hypothesized that the phosphine molecule would homolyze the thiyl radical to form an anomeric radical, which could then react with an olefin reactant to generate *C*-glycosides (Fig. 2B).

**Fig. 2:**
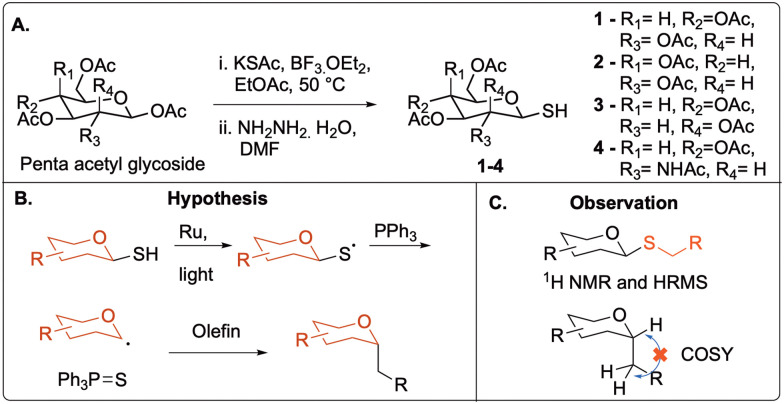
(A). Synthesis of 1-thiosugar donor molecules. (B). Hypothesis of the reaction. (C). Observation of the reaction.

Our first attempts used the 1-thiol derivative of glucose and allyl alcohol. Relative to published visible light thiol–ene reactions using aryl thiols and styrene acceptors, each of these substrates are more challenging for visible light photoredox catalysts to activate.^17,26^ Using 1-thiosugars at a concentration of 1 M in acetonitrile (MeCN) simply formed the disulfide of the starting material, even with phosphine, Ru(bpy)_3_ photocatalyst, and blue light activation conditions (Table 1, entry 1). A change of solvent from MeCN to dichloromethane (DCM) showed some desired activity, with 10% yield of a glycoside product (entry 2). This solvent switch encouraged us to optimize further to obtain the putative coupled product. To our surprise, characterization of the putative product using proton correlation spectroscopy (COSY) revealed that there was no correlation between the anomeric C1 protons and the protons from the incoming groups (Fig. 2C). Further high-resolution mass spectrometry (HRMS) and nuclear magnetic resonance (NMR) analysis confirmed that the glycoside product was an *S*-glycoside with 100% β-configuration, formed in what we presumed was a thiol–ene reaction. These unexpected results led us to investigate how a thiol–ene reaction occurred in the presence of a phosphine that is previously known to desulfurize thiols in similar conditions.^22–24^

**Table 1:** Optimization of *S*-glycoside reaction

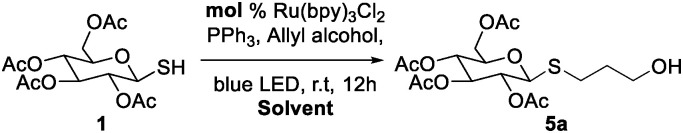					
#	Catalyst	PPh_3_ (equiv.)	Solvent^a^	5a yield (%)	Disulfide yield
1	10% Rubpy_3_·Cl_2_	1.5	MeCN (1 M)	—	80
2	10% Rubpy_3_·Cl_2_	1.5	DCM (1 M)	10	70
3	10% Rubpy_3_·Cl_2_	1.5	DCM (0.5 M)	61	25
4	10% Rubpy_3_·Cl_2_	1.5	DCM (0.1 M)	71	—
5	10% Rubpy_3_·Cl_2_	1.5	DCM (0.25 M)	85	—
6	10% Rubpy_3_·Cl_2_	1.5	CHCl_3_	20	—
7	10% Rubpy_3_·Cl_2_	1.5	Acetone	45	—
8	10% Rubpy_3_·Cl_2_	1.5	THF	34	—
9	10% Rubpy_3_·Cl_2_	1.5	MeOH	26	—
10	10% Rubpy_3_·Cl_2_	1.5	DMSO	48	—
11	10% Rubpy_3_·Cl_2_	1.5	H_2_O	90	—
**12**	**1% Rubpy**_**3**_ **·Cl**_**2**_	**1.5**	**H**_**2**_ **O**	**96**	—
13	2% Rubpy_3_·Cl_2_	1.5	H_2_O	92	—
14	5% Rubpy_3_·Cl_2_	1.5	H_2_O	91	—
15	1% Rubpy_3_·Cl_2_	0.5	H_2_O	65	—
16	1% Rubpy_3_·Cl_2_	1	H_2_O	74	—
17	1% Rubpy_3_·Cl_2_	3	H_2_O	81	—
18	1% Rubpy_3_·Cl_2_	1.5	H_2_O/MeOH (10:1)	69	—
19	1% Rubpy_3_·Cl_2_	1.5	H_2_O/MeOH (3:1)	51	—
20	1% Rubpy_3_·Cl_2_	1.5	H_2_O/MeOH (1:1)	40	—
21	1% Rubpy_3_·Cl_2_	1.5	H_2_O/MeOH (1:3)	34	—
22	1% Rubpy_3_·Cl_2_	1.5	H_2_O/MeOH (1:10)	29	—
23^b^	1% Rubpy_3_·Cl_2_	1.5	H_2_O	No reaction^b^	—

a Reactions were conducted at 0.25 M concentration of **1** unless noted.

b Reaction was conducted in the dark for 12 h at room temperature.

We tried various conditions to identify the optimal set for *S*-glycoside formation. Initially, we carried out optimization reactions by varying the solvent concentration in DCM. We observed a substantial increase in yield to 71% when the solvent concentration was reduced to 0.1 M (entry 4). Further optimization resulted in a yield of 85% at a concentration of 0.25 M (entry 5). However, the yield decreased when the concentration was 0.5 M. Evidently, the most favorable conditions for *S*-glycoside formation were achieved at a solvent concentration of 0.25 M. Using water boosted the yield to 90%, surpassing the yield with DCM at 10 mol% of ruthenium catalyst (entry 11). Additionally, the yield was further enhanced by conducting the reaction with just 1 mol% of ruthenium in H_2_O, resulting in 96% yield (entry 12). We did not observe *C*-glycoside formation under any Table 1 condition.

After optimizing the single-solvent system, we attempted optimization with a dual-solvent system to investigate potential for an “on-water effect”.^27,28^ Methanol alone yielded only a 26% yield (entry 9). Different ratios of water and methanol revealed a nearly linear effect on yield, where the yield of thiol–ene product decreased as the concentration of methanol in the reaction increased (entries 18–22). These results showed that water was the best solvent for this reaction.

The concentrations of catalyst and phosphine were both adjusted, with the optimal condition being 1% Ru(bpy)_3_ and 1.5 equivalents PPh_3_. In all conditions, triphenylphosphine oxide (TPPO) was observed, presumably because these reactions were performed in ambient, oxygen-containing conditions. Full conversion of excess PPh_3_ to TPPO was apparent by thin-layer chromatography. Substoichiometric PPh_3_ amounts (0.5 equiv.) led to reduced conversion at 65% yield (entry 15), significantly lower than 96% yield when PPh_3_ was used in excess (entry 12).

Subsequent trials with different photocatalysts explored *S*-glycoside formation at differing redox potentials. Comparing photocatalysts, photoexcited Ru*(bpy)_3_ with its *E*_ox_ = +0.77 V (*vs.* saturated calomel electrode (SCE)) is only weakly reactive with thiols on its own.^21^ For direct thiyl radical formation, a stronger photooxidant like Ru*(bpz)_3_ with its *E*_ox_ = +1.35 V (*vs.* SCE) or Ru*(bpm)_3_ with *E*_ox_ = +1.21 (*vs.* SCE) is typically required.^17,26^ However, in our conditions, this reactivity was reversed, with Rubpy being superior to Rubpz, Rubpm, and iridium-based photocatalysts (Table 2, entries 1–5).

**Table 2:** Catalyst and phosphine activator scope under optimized conditions

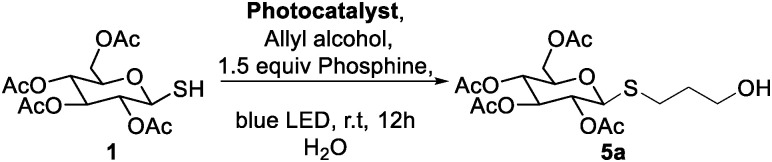				
#	Catalyst	Phosphine^a^	Solvent^b^	5a yield
1	(1 mol%) Rubpy_3_·Cl_2_	PPh_3_	H_2_O	96%
2	1% Ru(bpz)_3_·(PF_6_)_2_	PPh_3_	H_2_O	67%
3	1% Ru(bpm)_3_·PF_6_	PPh_3_	H_2_O	56%
4	(1%) *fac*-tris(2-phenylpyridine)iridium	PPh_3_	H_2_O	58%
5	Ir[dF(CF_3_)ppy]_2_(dtbbpy)·PF_6_	PPh_3_	H_2_O	71%
6	1% Rubpy_3_·Cl_2_	TCEP	H_2_O	45%
7	1% Rubpy_3_·Cl_2_	TOP	H_2_O	Trace
8	1% Rubpy_3_·Cl_2_	TBP	H_2_O	23%
9	1% Rubpy_3_·Cl_2_	TOP/TOPO (1 : 1)	H_2_O	Trace
10	1% Rubpy_3_·Cl_2_	PPh_3_O	H_2_O	—

a Reactions conducted with 1.5 equivalents of phosphine.

b Reactions conducted at 0.25 M concentration of 1. TCEP = tris(carboxyethyl)phosphine. TOP = trioctylphosphine. TBP = tributylphosphine. TOPO = trioctylphosphine oxide.

Other phosphine species including tris(carboxyethyl)phosphine (TCEP), tributyl phosphine (TBP), and trioctyl phosphine (TOP) afforded lower yields of thiol–ene product relative to triphenylphosphine. The redox potential of PPh_3_ is known to be +1.0 V *vs.* SCE,^29^ slightly above Ru*(bpy)_3_ of +0.77 V (*vs.* SCE). TCEP has a much lower redox potential of −0.29 V.^30^ TBP and TOP do not have readily available published redox potentials, but being alkyl phosphines like TCEP are likely to be lower than the +1.0 V of PPh_3_. We observed that closely matching the redox potential between the Rubpy oxidant and PPh_3_ gave the best results (Table 2, entry 1) relative to lower redox phosphines like TCEP (entry 6) or higher redox Ru species like Rubpz (entry 2). Triphenylphosphine oxide (TPPO) alone did not yield any conversion (entry 10), indicating the PPh_3_ was the active species. Redox mediator effects have been observed between the highly oxidizing Rubpz, *p*-toluidine, and thiols,^26^ but not, to our knowledge, with Rubpy as photocatalyst.

After optimization, the substrate scope was briefly explored. Five olefins not typically reactive in visible light thiol–ene conditions were chosen.^17,26^ Glucose and galactose thiosugars gave the desired products in good to excellent yields (Scheme 1). Allyl alcohol was used in the initial reaction optimization. In addition to alcohols, these reaction conditions were compatible with carbonates, amides, and esters, indicating a useful chemoselectivity for this radical reaction. Addition to a model dehydroalanine amino acid mimic suggest that these aqueous conditions may be used in peptide and protein-based dehydroalanine modification reactions.^31,32^

**Scheme 1:**
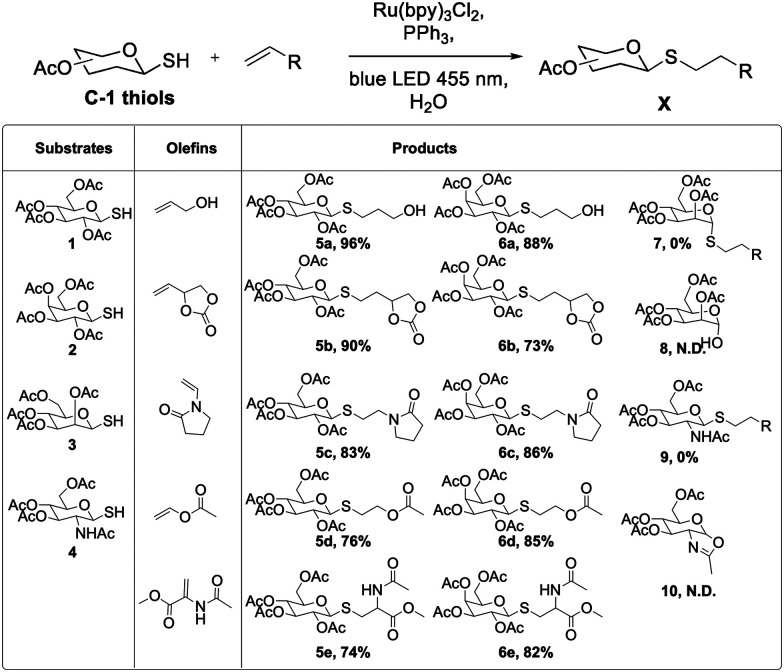
Substrate scope under optimized phosphine-accelerated photo-thiol–ene conditions.

After success with glucose and galactose sugars, however, mannose and *N*-acetylglucosamine (GlcNAc) derivatives did not yield the expected thioether products. The mannose derivative formed the hemiacetal 8, and the GlcNAc compound formed the oxazoline 10, each resulting from intramolecular attack on the sugar ring. These two substrates are primed for anchimeric assistance from the 2-acetyl or 2-amido groups, suggesting a different reactivity pattern when such intramolecular reactions are especially favorable.

To gain insight into the reaction mechanism under phosphine acceleration, various control reactions were conducted (Table 3). Controls that lacked each component separately, the metal catalyst, phosphine reagent, or light showed no conversion (Table 3, entries 1–4). Air was also demonstrated to be important for conversion, because dry DCM or H_2_O under argon atmosphere failed to afford the *S*-glycosylation product (entries 11 and 12). Use of 1.5 equivalents of the radical trap (2,2,6,6-tetramethylpiperidin-1-yl)oxyl (TEMPO) only yielded a trace amount of thiol–ene product in water, and no product when TEMPO was added in DCM (entries 13 and 14).

**Table 3:** Controls for phosphine-accelerated thiol–ene reaction

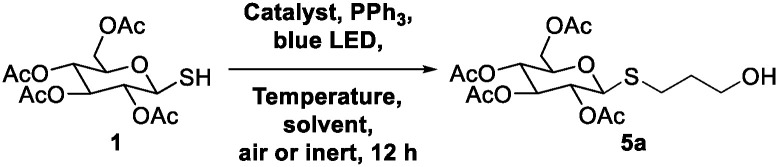					
#	Catalyst	PPh_3_ (equiv.)	Solvent^a^	5a yield (%)	Condition notes
1	(1 mol%) Rubpy_3_·Cl_2_	1.5	H_2_O	96	—
2^b^	1% Rubpy_3_·Cl	1.5	H_2_O	—	Dark
3^c^	1% Rubpy_3_·Cl_2_	—	H_2_O	Trace	—
4^c^	1% Rubpy_3_·Cl_2_	—	DCM	—	—
5^c^	—	1.5	H_2_O	—	—
6^c^	—	1.5	DCM	—	—
7	1% Rubpy_3_·Cl_2_	1.5	Toluene	75	—
8^d^	1% Rubpy_3_·Cl_2_	—	Toluene^d^	—	Dark, 60 °C
9^d^	—	1.5	Toluene^d^	—	Dark, 60 °C
10^d^	1% Rubpy_3_·Cl_2_	1.5	Toluene^d^	60	Dark, 60 °C
11^e^	1% Rubpy_3_·Cl_2_	1.5	DCM^e^	-—	Degassed
12^e^	1% Rubpy_3_·Cl_2_	1.5	H_2_O^e^	Trace	Degassed
13^f^	1% Rubpy_3_·Cl_2_	1.5	H_2_O^f^	Trace	TEMPO
14^f^	1% Rubpy_3_·Cl_2_	1.5	DCM^f^	—	TEMPO

a Reactions conducted at 0.25 M concentration of 1.

b Reaction conducted in the dark at r.t. for 12 h.

c Reaction conducted without either PPh_3_ or Ru(bpy)_3_ catalyst.

d Reaction conducted in the dark at 60 °C.

e Reaction conducted in inert DCM purged with argon.

f Reaction conducted with 1.5 equiv. (2,2,6,6-tetramethylpiperidin-1-yl)oxyl (TEMPO) under standard conditions.

This thiol–ene reaction was also attempted under heating conditions as an alternative to photochemical Rubpy activation. For these studies, we used toluene for its ability to fully dissolve reactants and to withstand heating to 60 °C (Table 3, entries 8–10). The standard phosphine-accelerated thiol–ene condition in toluene gave the expected product in 75% yield. The alternative heat-based activation was attempted with 60 °C heating in the dark. Remarkably, the phosphine and Ru(bpy)_3_ condition afforded a similar 60% yield to the photoredox/room temperature conditions. Both the phosphine and Ru(bpy)_3_ catalyst were required for heat-based conversion (entry 10). Triphenylphosphine oxide was also observed to form as a byproduct of the heated condition. Together, these studies suggested that a radical mechanism involving Ru(bpy)_3_, phosphine, oxygen, and thiol was evident, rather than simple pyrolysis or substitution from a thiol-phosphine adduct.

Through control experiments and literature, we proposed a mechanism involving photoexcitation of the Ru(bpy)_3_ catalyst and oxidation of triphenylphosphine to the radical cation species (Fig. 3). The oxidized triphenylphosphine may then act as a redox mediator to oxidize the thiol to the thiyl radical, which reacts with the olefin. This mediating reaction was necessary in these conditions because photoexcited Ru*(bpy) had insufficient *E*_ox_ to efficiently oxidize thiols.^17,26^ Capture of the resulting thiol–ene radical species by another thiol then initiated a final catalytic cycle. The observation that air (O_2_) was required for turnover and the generation of triphenylphosphine oxide suggested the involvement of triphenylphosphine as a sacrificial redox mediator in this reaction. Use of 0.5 equivalents of PPh_3_ led to lower yield, indicating that catalysis is possible but inefficient for PPh_3_ (see Table 1, entry 15). The complete β-selectivity from β-glucose and β-galactose substrates 1 and 2 to their respective β-thiosugar products also agreed with a radical pathway.

**Fig. 3:**
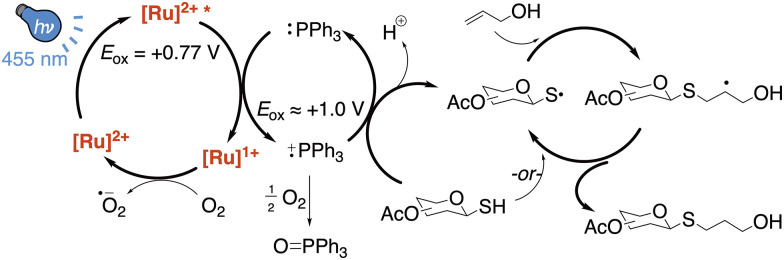
Plausible mechanistic pathway for the phosphine-accelerated Ru(bpy)_3_-catalyzed radical thiol–ene reaction.

In this brief report, we focus on aliphatic olefins and acetyl-protected sugars, but we expect the substrate scope is broader. We will be exploring further applications of these identified conditions, including reactions with aromatic olefins, alternatively protected sugars, and more challenging peptide acceptors. Our results suggest that the reaction progresses through a series of radical catalytic cycles, and these mild thiol–ene conditions are promising for the formation of new series of substituted 1-thiosugars and, by extension, their oxidized products for medicinal and chemical utility.

## Conflicts of interest

There are no conflicts to declare.

## Supplementary Material

CC-061-D5CC00131E-s001

## Data Availability

The data supporting this article have been included as part of the ESI.†
