# Supplementary material for: Thioglycoside functionalization via chemoselective phosphine acceleration of a photocatalytic thiol–ene reaction
Source: Chem Commun (Camb). 2025 Apr 29;61(52):9484–7. doi: 10.1039/d5cc00131e (PMC12128037; doi:10.1039/d5cc00131e)
Supplement: CC-061-D5CC00131E-s001 [file CC-061-D5CC00131E-s001.pdf]

Supplementary Information (SI) for ChemComm.
This journal is © The Royal Society of Chemistry 2025

Supporting Information

Thioglycoside functionalization via chemoselective phosphine acceleration of a photocatalytic thiol-ene reaction

Table of Contents

Section		Page
1	General information	3
2	Experimental procedures	3
	2.1 General procedure for synthesis of 1-thiol glycosyl donors (**1-4**):	3
	2.2 General procedure for phosphine-accelerated photo-thiol-ene reaction	3
	2.3 Procedure for radical trapping experiment	4
	2.4 Condition optimization table	5
3	Analytical data for synthesized compounds	6
	3.1 ^{1}H and ^{13}C-NMR spectra of compound **5a**	9
	3.2 ^{1}H and ^{13}C-NMR spectra of compound **5b**	10
	3.3 ^{1}H and ^{13}C-NMR spectra of compound **5c**	11
	3.4 ^{1}H and ^{13}C-NMR spectra of compound **5d**	12
	3.5 ^{1}H and ^{13}C-NMR spectra of compound **5e**	13
	3.6 ^{1}H and ^{13}C-NMR spectra of compound **6a**	14
	3.7 ^{1}H and ^{13}C-NMR spectra of compound **6b**	15
	3.8 ^{1}H and ^{13}C-NMR spectra of compound **6c**	16
	3.9 ^{1}H and ^{13}C-NMR spectra of compound **6d**	17
	3.10 ^{1}H and ^{13}C-NMR spectra of compound **6e**	18
4	Supplementary Reference	19

General information

All reactions were conducted under ambient conditions using undistilled solvents until otherwise stated. Reagents were purchased at the highest commercial quality and used without further purification. Triphenylphosphine was purchased from TCI Chemicals and utilized in its powdered form for the reactions. The photochemical reactions were conducted in an Efficiency Aggregators BPR200 Biophotoreactor equipped with blue (455 nm) LED lights and illumination power set at 100% intensity. Silica gel TLC plates were used to monitor the reactions with short-wavelength ultraviolet light to visualize UV-vis active spots and charring after spraying with 15% sulfuric acid in ethanol (EtOH) to visualize sugars. Column chromatography was performed on 230−400 mesh silica gel. Nuclear magnetic resonance (NMR) spectroscopy was performed on a Bruker Avance NEO 500 MHz instrument. ^{1}H NMR spectra were recorded at 500 MHz, chemical shifts are given in parts per million, and coupling is constant in Hertz. ^{13}C NMR spectra were recorded at 125 MHz. High resolution electrospray ionization mass spectrometry (HR-ESI-MS) analysis was performed using an electrospray ionization technique using a Thermo LTQ Orbitrap XL analyzer, with ions given in m/z.

Experimental Procedures

General procedure for synthesis of 1-thiol donors (1-4):

To a solution of per-O-acetyl glycoside (2000 mg) in ethyl acetate (30 mL), potassium thioacetate was added (3 equiv.) and stirred for 2 minutes. After adding $BF_3·Et_2O$ (4 equiv.) to the mixture, the reaction proceeded at 50 °C for 4 h. The reaction was monitored using TLC for the complete conversion of the starting material. Afterward, the mixture was treated with triethylamine (TEA) and filtered to remove potassium salts. The resulting reaction mixture was concentrated under reduced pressure, and the residue was dissolved in dimethylformamide (DMF) (10 mL). To this solution, hydrazine hydrate ($N_2H_4·H_2O$, 1.5 equiv) was added, and the reaction was allowed to proceed at room temperature for 30 minutes. The reaction mixture was then neutralized with acetic acid and extracted with dichloromethane (CH_2Cl_2) and water. The combined organic phases were dried over magnesium sulfate ($MgSO_4$) and concentrated under reduced pressure. Finally, the crude material was purified by silica gel chromatography, yielding compounds **1-4**. All starting

materials were synthesized according to the reported literature and confirmed by NMR analysis.[1]

General procedure for synthesis of thioglycoside product:

To a solution of anomeric thiol (50 mg, 1 equiv) in water (0.25 M), olefin (2 to 3 equiv), powdered PPh_3 (54 mg, 1.5 equiv), and tris(2,2'-bipyridyl)dichlororuthenium(II) hexahydrate ($Ru(bpy)_3$) catalyst (1 mol%) were added. The mixture was allowed to irradiate under blue LED 455 nm conditions with stirring for 12 hours. The reaction progress was monitored by TLC. After complete consumption of the starting material (typically by 12 h), the reaction mixture was diluted with DCM (10 mL), and saturated aqueous NH_4Cl solution (5 mL) was added. The organic layer was dried over anhydrous Na_2SO_4, concentrated, and purified by column chromatography using hexane/EtOAc as eluent to afford the corresponding S-glycoside product. Notably, flaked PPh_3 gives lower conversion than powder form of this reagent.

Procedure for radical trapping experiment:

To a solution of anomeric thiol (50 mg, 1 equiv), in water or DCM (0.25 M), olefin (2 equiv), PPh_3 (54 mg, 1.5 equiv, [powdered not flakes]), TEMPO (32 mg,1.5 equiv) and $Ru(bpy)_3$ catalyst (1 mol%) were added and allowed to irradiate at the blue LED 455 nm and stirred. After 12 hours, the reaction was monitored by TLC. No observation of the product was observed in the DCM condition. A trace amount of product was observed in the water condition, but was not isolated.

Procedure for phosphine- or catalyst-dependence experiments:

To a solution of anomeric thiol (50 mg,1 equiv), in water or DCM (0.25 M), olefin (2 equiv), and $Ru(bpy)_3$ catalyst (1 mol%) were added and allowed to irradiate at the blue LED 455 nm and stirred. After 12 hours, the reaction was monitored by TLC. Similarly, a solution of anomeric thiol (1 equiv), in water or DCM (0.25 M), olefin (2 equiv), and PPh_3 (1.5 equiv, [powdered not flakes]) were added and allowed to irradiate at the blue LED 455 nm and stirred. After 12 hours, the reaction was monitored by TLC. No observation of the product

was observed in any condition without both phosphine and $Ru(bpy)_3$, either in water or DCM.

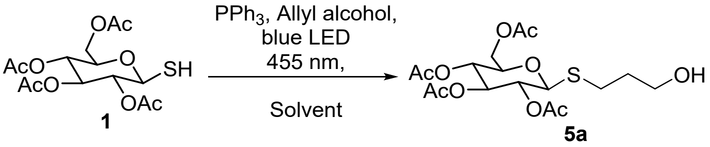

S. No.	Catalyst	PPh_3 (Equiv)	Solvent	3a Yield	Disulfide yield
1	10 % $Rubpy_3Cl_2$	1.5	ACN (1 M)	-	80%
2	10 % $Rubpy_3Cl_2$	1.5	DCM (1 M)	10%	70%
3	10 % $Rubpy_3Cl_2$	1.5	DCM (0.5 M)	61%	25%
4	10 % $Rubpy_3Cl_2$	1.5	DCM (0.1 M)	71 %	-
5	10 % $Rubpy_3Cl_2$	1.5	DCM (0.25 M)	85 %	-
6	10 % $Rubpy_3Cl_2$	1.5	$CHCl_3$	20 %	-
7	10 % $Rubpy_3Cl_2$	1.5	Acetone	45 %	-
8	10 % $Rubpy_3Cl_2$	1.5	THF	34 %	-
9	10 % $Rubpy_3Cl_2$	1.5	MeOH	26 %	-
10	10 % $Rubpy_3Cl_2$	1.5	DMSO	48%	-
11	10 % $Rubpy_3Cl_2$	1.5	H_2O	90 %	-
12	10 % $Rubpy_3Cl_2$	-	H_2O	Trace	-
13	10 % $Rubpy_3Cl_2$	-	DCM	-	-
14	-	1.5	H_2O	-	-
15	-	1.5	DCM	-	-
**16**	**(1 mol %) $Rubpy_3Cl_2$**	**1.5**	**H_2O**	**96 %**	-
17	(2 mol %) $Rubpy_3Cl_2$	1.5	H_2O	92 %	-
18	(5 mol %) $Rubpy_3Cl_2$	1.5	H_2O	91 %	-
19	1 % $Rubpy_3Cl_2$	0.5	H_2O	65 %	-
20	1 % $Rubpy_3Cl_2$	1	H_2O	74 %	-
21	1 % $Rubpy_3Cl_2$	3	H_2O	81 %	-
22	1 % $Rubpy_3Cl_2$	1.5	H_2O/MeOH (10:1)	69 %	-
23	1 % $Rubpy_3Cl_2$	1.5	H_2O/MeOH (3:1)	51 %	-
24	1 % $Rubpy_3Cl_2$	1.5	H_2O/MeOH (1:1)	40 %	-
25	1 % $Rubpy_3Cl_2$	1.5	H_2O/MeOH (1:3)	34 %	-
26	1 % $Rubpy_3Cl_2$	1.5	H_2O/MeOH (1:10)	29 %	-
27	1 % $Rubpy_3Cl_2$	1.5	Toluene	75 %	-
28	1 % $Rubpy_3Cl_2$	-	Toluene	-	-
29	-	1.5	Toluene	-	-
30	1 % $Rubpy_3Cl_2$	1.5	Toluene (60 ˚C)	60 %	-
31	1 % $Rubpy_3Cl$	1.5	H_2O (Dark)	0	-
32	1 % $Ru(bpz)_3(PF_6)_2$	1.5	H_2O	67 %	-
33	1 % $Rubpm_3PF_6$	1.5	H_2O	56 %	-
34	(1 %) fac-Tris(2-phenylpyridine)iridium	1.5	H_2O	58 %	-
35	$Ir[dF(CF_3)ppy]_2(dtbbpy)PF_6$	1.5	H_2O	71 %	-
36	1 % $Rubpy_3Cl_2$	TCEP	H_2O	45%	-
37	1 % $Rubpy_3Cl_2$	TOP	H_2O	Trace	-
38	1 % $Rubpy_3Cl_2$	TBP	H_2O	23%	-
39	1 % $Rubpy_3Cl_2$	TOP/TOPO	H_2O	Trace	-
40	1 % $Rubpy_3Cl_2$	PPh_3O	H_2O	-	-
41	1 % $Rubpy_3Cl_2$	PPh_3	H_2O	Trace	-

Analytical data for synthesized compounds

(2R,3R,4S,5R,6S)-2-(acetoxymethyl)-6-((3-hydroxypropyl)thio)tetrahydro-2H-pyran-3,4,5-triyl triacetate (5a): (Colorless liquid, 96%, Hexane/EtOAc = 60:40). ^{1}H NMR (500 MHz, CDCl3) δ 5.16 (t, *J* = 9.5 Hz, 1H), 5.00 (m, 2H), 4.42 (d, *J* = 10.0 Hz, 1H), 4.17 (dd, *J* = 12.5, 5 Hz, 1H), 4.10 (dd, *J* = 12.45, 2.5 Hz, 1H), 3.70 – 3.62 (m, 3H), 2.70 (m, 1H), 2.64 (m, 1H), 2.02 (s, 3H), 2.00 (s, 3H), 1.96 (s, 3H), 1.94 (s, 3H), 1.80 – 1.74 (m, 2H). ^{13}C NMR (126 MHz, $CDCl_3$) δ 170.66, 170.15, 169.55, 169.40, 83.53, 76.04, 73.80, 69.74, 68.32, 62.06, 60.56, 32.05, 26.11, 20.72, 20.71, 20.60, 20.57. HR-ESI-MS $[M + Na]^+$ $C_{17}H_{26}NaO_{10}S$ calcd. for m/z 445.1144, found 445.1130.

(2R,3R,4S,5R,6S)-2-(acetoxymethyl)-6-((2-(2-oxo-1,3-dioxolan-4-yl)ethyl)thio)tetra hydro-2H-pyran-3,4,5-triyl triacetate (5b): (Off white sticky solid, 90%, Hexane/EtOAc = 70:30). ^{1}H NMR (500 MHz, $CDCl_3$) δ 5.05 – 4.98 (m, 1H), 4.95 – 4.76 (m, 1H), 4.52 (m, 1H), 4.43 (m, 1H), 4.23 – 4.01 (m, 3H), 3.69 – 3.61 (m, 1H), 2.99 – 2.85 (m, 1H), 2.77 (m, 1H), 2.64 (m, 1H), 2.02 (s, 3H), 1.99 (s, 3H), 1.97 (s, 3H), 1.94 (s, 3H). ^{13}C NMR (126 MHz, $CDCl_3$) δ 170.50, 170.07, 169.44, 169.39, 154.64, 154.62, 83.82, 83.53, 76.30, 76.16, 75.29, 75.25, 73.69, 73.62, 69.73, 69.38, 69.18, 69.17, 68.20, 68.16, 61.88, 61.80, 34.38, 34.37, 25.49, 25.15, 20.71, 20.67, 20.57, 20.56. HR-ESI-MS $[M + Na]^+$ $C_{19}H_{26}NaO_{12}S$ calcd. for m/z 501.1043, found 501.1021.

(2R,3R,4S,5R,6S)-2-(acetoxymethyl)-6-((2-(2-oxopyrrolidin-1-yl)ethyl)thio)tetrahydro-2H-pyran-3,4,5-triyl triacetate (5c): (Colorless liquid, 83%, Hexane/EtOAc = 70:30). ^{1}H NMR (500 MHz, $CDCl_3$) δ 5.16 (t, *J* = 9.4 Hz, 1H), 4.99 (m, 2H), 4.49 (d, *J* = 10.0 Hz, 1H), 4.18 (dd, *J* = 12.5, 5 Hz, 1H), 4.09 (dd, *J* = 12, 2.5 Hz, 1H), 3.68 (m, 1H), 3.54 – 3.42 (m, 1H), 3.43 – 3.32 (m, 4H), 2.84 (m, 1H), 2.71 (m, 1H), 2.31 (m, 2H), 2.01 (s, 3H), 1.99 (s, 3H), 1.96 (s, 3H), 1.94 (s, 3H). ^{13}C NMR (126 MHz, $CDCl_3$) δ 175.10, 170.57, 170.08, 169.43, 83.45, 76.01, 73.80, 69.82, 68.28, 62.07, 47.78, 42.56, 30.83, 27.95, 20.73, 20.68, 20.58, 20.57, 18.36, 18.09. HR-ESI-MS $[M + Na]^+$ $C_{20}H_{29}NNaO_{10}S$ calcd. for m/z 498.1410, found 498.1401.

(2S,3R,4S,5R,6R)-2-((2-acetoxyethyl)thio)-6-(acetoxymethyl)tetrahydro-2H-pyran-3,4,5-triyl triacetate (**5d**): (Colorless sticky solid, 76%, Hexane/EtOAc = 70:30). ^{1}H NMR (500 MHz, $CDCl_3$) δ 5.16 (t, *J* = 9.5 Hz, 1H), 4.99 (m, 2H), 4.48 (d, *J* = 10.0 Hz, 1H), 4.25 – 4.12 (m, 3H), 4.08 (dd, *J* = 12.5, 2.5 Hz, 1H), 3.66 (m, 1H), 2.91 (m, 1H), 2.74 (m, 1H), 2.02 (s, 3H), 1.99 (m, 6H), 1.96 (s, 3H), 1.94 (s, 3H). ^{13}C NMR (126 MHz, $CDCl_3$) δ 169.63, 169.59, 169.12, 168.36, 82.63, 75.01, 72.74, 68.77, 67.24, 62.59, 61.05, 27.71, 19.83, 19.66, 19.58, 19.56. HR-ESI-MS [M + Na]$^+$ $C_{18}H_{26}NaO_{11}S$ calcd. for m/z 473.1094, found 473.1081.

(2S,3R,4S,5R,6R)-2-((2-acetamido-3-methoxy-3-oxopropyl)thio)-6-(acetoxymethyl)tetrahydro-2H-pyran-3,4,5-triyl triacetate (**5e**): (Colorless liquid, 74%, EtOAc = 100). ^{1}H NMR (500 MHz, $CDCl_3$) δ 6.45 (m, 1H), 5.15 (m, 1H), 4.99 (m, 1H), 4.91 (m, 1H), 4.74 (m, 1H), 4.45 (m, 1H), 4.21 – 4.07 (m, 2H), 3.72 – 3.61 (m, 4H), 3.14 (m, 1H), 2.99 (m, 1H), 2.04 (s, 3H), 2.00 – 1.40 (m, 12H). ^{13}C NMR (126 MHz, $CDCl_3$) δ 170.92, 170.66, 170.62, 170.60, 170.08, 170.06, 169.93, 169.87, 169.48, 169.44, 169.38, 169.34, 83.73, 83.35, 76.20, 76.08, 73.55, 69.92, 69.75, 68.13, 68.09, 62.15, 61.86, 52.75, 52.70, 52.28, 51.85, 32.45, 31.75, 22.94, 20.71, 20.69, 20.57, 20.56. HR-ESI-MS [M + Na]$^+$ $C_{20}H_{29}NNaO_{12}S$ calcd. for m/z 530.1308, found 530.1288.

(2R,3S,4S,5R,6S)-2-(acetoxymethyl)-6-((3-hydroxypropyl)thio)tetrahydro-2H-pyran-3,4,5-triyl triacetate (**6a**): (Colorless liquid, 88%, Hexane/EtOAc = 60:40). ^{1}H NMR (500 MHz, $CDCl_3$) δ 5.37 (dd, *J* = 3.5, 1.5 Hz, 1H), 5.20 (t, *J* = 10.0 Hz, 1H), 4.98 (dd, *J* = 10.0, 3.5 Hz, 1H), 4.41 (d, *J* = 9.9 Hz, 1H), 4.04 (m, 1H), 3.68 (t, *J* = 6 Hz, 2H), 2.82 (m, 1H), 2.75 (m, 1H), 2.09 (s, 3H), 2.00 (s, 6H), 1.92 (s, 3H), 1.79 (m, 2H). ^{13}C NMR (126 MHz, $CDCl_3$) δ 170.44, 170.20, 170.06, 169.80, 84.07, 74.65, 71.86, 67.34, 67.08, 61.58, 60.66, 32.04, 26.28, 20.84, 20.68, 20.59 HR-ESI-MS [M + Na]$^+$ $C_{17}H_{26}NaO_{10}S$ calcd. for m/z 445.1144, found 445.1131.

(2R,3S,4S,5R,6S)-2-(acetoxymethyl)-6-((2-(2-oxo-1,3-dioxolan-4-yl)ethyl)thio)tetrahydro - 2H-pyran-3,4,5-triyl triacetate (**6b**): (off white solid, 73%, Hexane/EtOAc = 70:30). ^{1}H NMR (500 MHz, $CDCl_3$) δ 6.24 (d, *J* = 4 Hz, 1H), 5.43 – 5.35 (m, 3H), 5.20 – 5.08 (m, 3H), 4.98 (dt, *J* = 10.0, 3.5 Hz, 1H), 4.89 – 4.79 (m, 1H), 4.51 (m, 2H), 4.40 (m, 1H), 4.21 (m, 1H), 4.14 – 3.97 (m, 10H),

3.91 – 3.84 (m, 2H), 2.93 (m, 1H), 2.78 (m, 2H), 2.66 (m, 1H), 2.12 (s, 3H), 2.10 (s, 6H), 2.08 (s, 3H), 2.00 (s, 3H), 1.99 (s, 3H), 1.97 (s, 3H), 1.92 (s, 3H). ^{13}C NMR (126 MHz, $CDCl_3$) δ 170.38, 170.03, 169.67, 154.61, 91.91, 83.85, 75.37, 74.94, 71.67, 70.44, 69.12, 68.71, 67.51, 66.68, 66.14, 61.26, 34.40, 20.98, 20.78, 20.68, 20.66, 20.60, 20.57. HR-ESI-MS $[M + Na]^+$ $C_{19}H_{26}NaO_{12}S$ calcd. for m/z 501.1043, found 501.1023.

(2R,3S,4S,5R,6S)-2-(acetoxymethyl)-6-((2-(2-oxopyrrolidin-1-yl)ethyl)thio)tetrahydro-2H-pyran-3,4,5-triyl triacetate (**6c**): (Colorless waxy solid, 86%, Hexane/EtOAc = 70:30). ^{1}H NMR (500 MHz, $CDCl_3$) δ 5.37 (dd, *J* = 3.5, 1.0 Hz, 1H), 5.17 (t, *J* = 9.5 Hz, 1H), 4.99 (dd, *J* = 10.0, 3.5 Hz, 1H), 4.48 (d, *J* = 10 Hz, 1H), 4.07 (m, 2H), 3.50 (m, 1H), 3.45 – 3.32 (m, 4H), 2.87 (m, 1H), 2.76 – 2.67 (m, 1H), 2.28 (m, 4H), 2.10 (s, 3H), 2.00 (s, 3H), 1.98 (s, 3H), 1.92 (s, 3H). ^{13}C NMR (126 MHz, $CDCl_3$) δ 175.06, 170.39, 170.19, 169.99, 169.65, 83.84, 74.58, 71.81, 67.28, 67.08, 61.44, 47.70, 42.60, 30.82, 27.88, 20.86, 20.79, 20.67, 20.57, 18.07 HR-ESI-MS $[M + H]^+$ $C_{20}H_{30}NO_{10}S$ calcd. for m/z 479.1590, found 476.1572.

(2S,3R,4S,5S,6R)-2-((2-acetoxyethyl)thio)-6-(acetoxymethyl)tetrahydro-2H-pyran-3,4,5-triyl triacetate (**6d**): (Colorless waxy solid, 85%, Hexane/EtOAc = 70:30). ^{1}H NMR (500 MHz, $CDCl_3$) δ 5.37 (dd, *J* = 3.5, 1.5 Hz, 1H), 5.17 (t, *J* = 10.0 Hz, 1H), 4.98 (dd, *J* = 10.0, 3.5 Hz, 1H), 4.46 (d, *J* = 9.5 Hz, 1H), 4.20 (m, 2H), 4.07 (m, 2H), 3.88 (m, 1H), 2.94 (m, 1H), 2.76 (m, 1H), 2.10 (s, 3H), 2.06 – 1.95 (m, 9H), 1.92 (s, 3H). ^{13}C NMR (126 MHz, $CDCl_3$) δ 170.60, 170.40, 170.21, 170.02, 169.58, 84.15, 74.64, 71.82, 67.25, 67.14, 63.62, 61.49, 28.80, 20.84, 20.77, 20.65, 20.63, 20.57 HR-ESI-MS $[M + Na]^+$ $C_{18}H_{26}NaO_{11}S$ calcd. for m/z 473.1094, found 473.1076.

(2S,3R,4S,5S,6R)-2-((2-acetamido-3-methoxy-3-oxopropyl)thio)-6-(acetoxymethyl)tetra hydro-2H-pyran-3,4,5-triyl triacetate (**6e**): (Colorless oily liquid, 82%, EtOAc = 100). ^{1}H NMR (500 MHz, $CDCl_3$) δ 6.39 (m, 1H), 5.37 (m, 1H), 5.12 (m, 1H), 4.97 (m, 1H), 4.75 (m, 1H), 4.42 (m, 1H), 4.12 – 4.04 (m, 2H), 3.88 (m, 1H), 3.70 (d, *J* = 1.0 Hz, 3H), 3.16 (m, 1H), 3.00 (m, 1H), 2.10 (s, 3H), 2.02 – 1.97 (m, 9H), 1.92 (s, 3H). ^{13}C NMR (126 MHz, $CDCl_3$) δ 170.97, 170.64,

170.42, 170.36, 170.16, 169.97, 169.87, 169.77, 169.69, 84.28, 83.62, 74.90, 74.69, 71.66, 71.64, 67.42, 67.20, 67.17, 66.93, 61.76, 61.41, 52.75, 52.68, 52.30, 51.81, 32.64, 31.58, 23.01, 22.98, 20.80, 20.65, 20.55 HR-ESI-MS $[M + Na]^+$ $C_{20}H_{29}NNaO_{12}S$ calcd. for m/z 530.1308, found 530.1287.

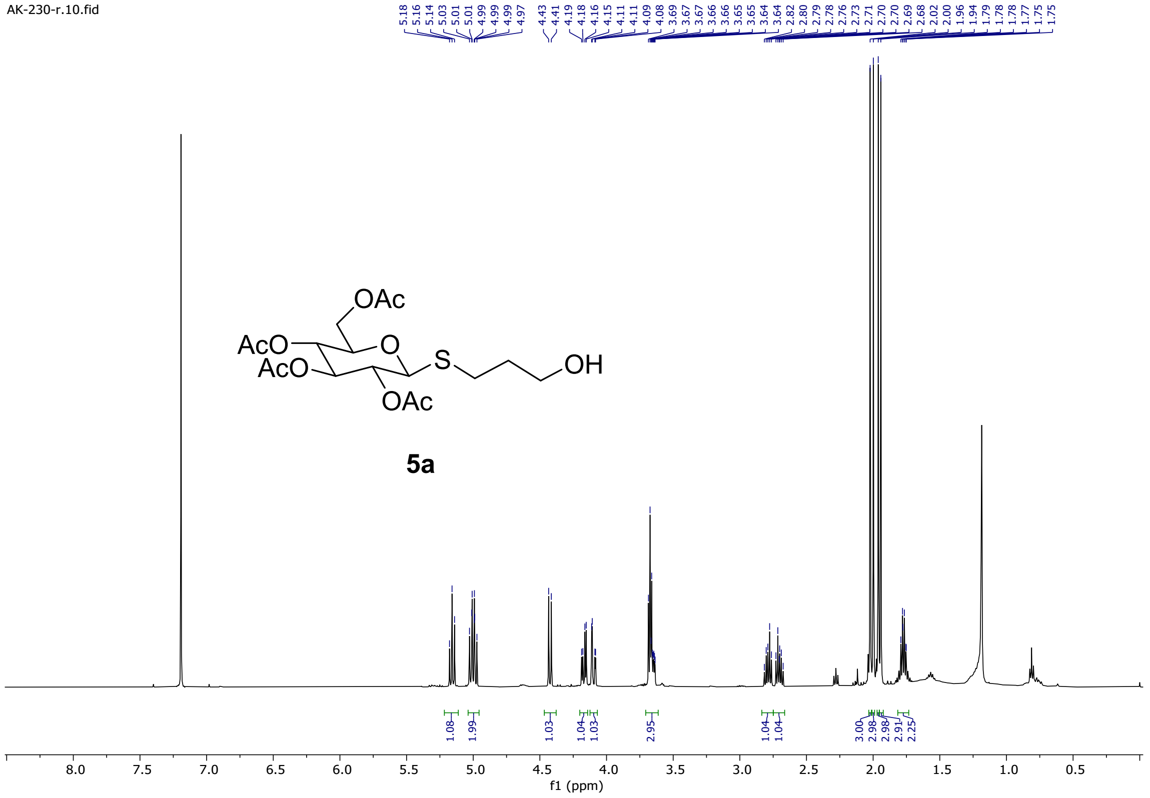

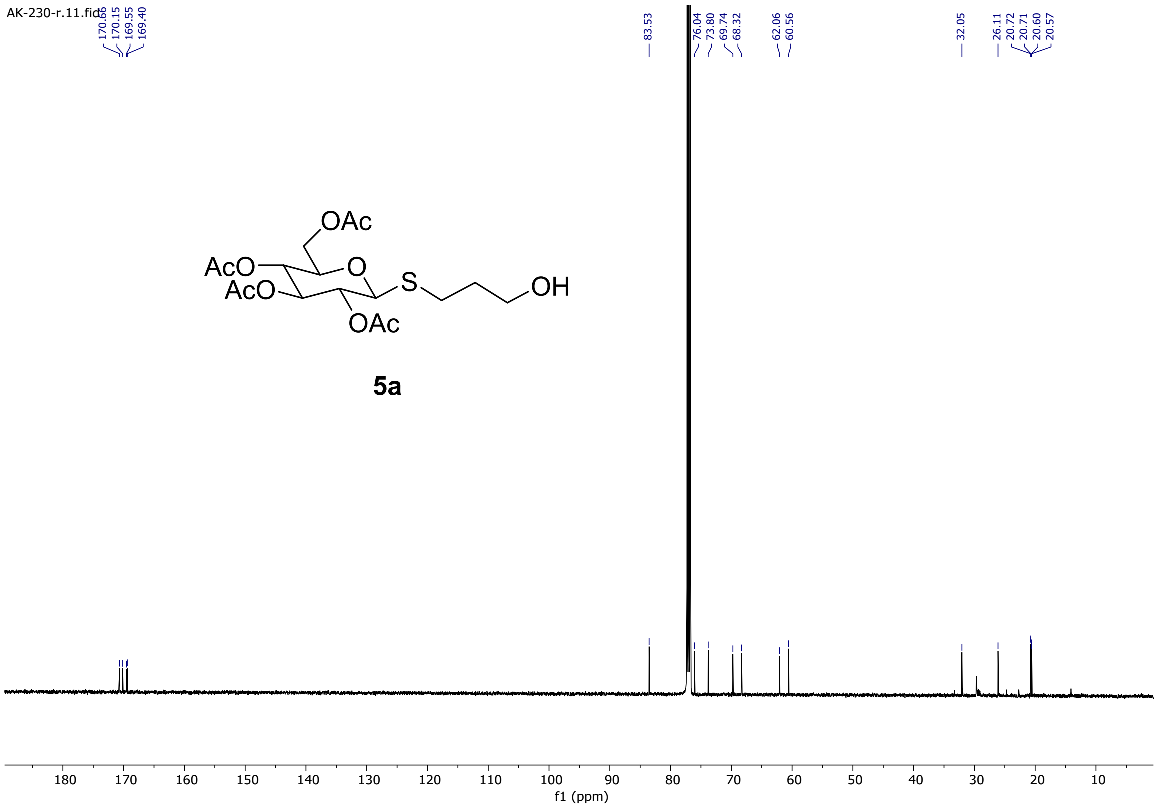

^{1}H and ^{13}C-NMR spectra of compound **5a**

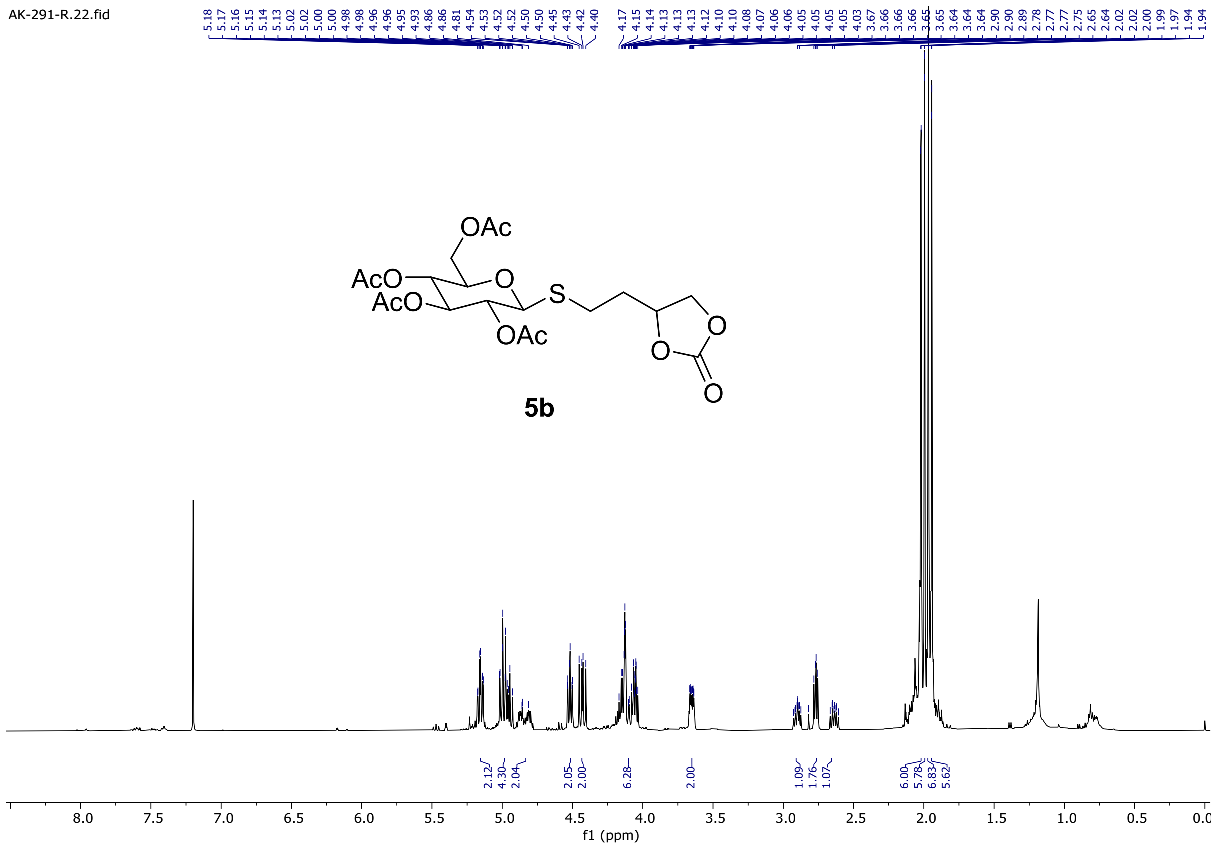

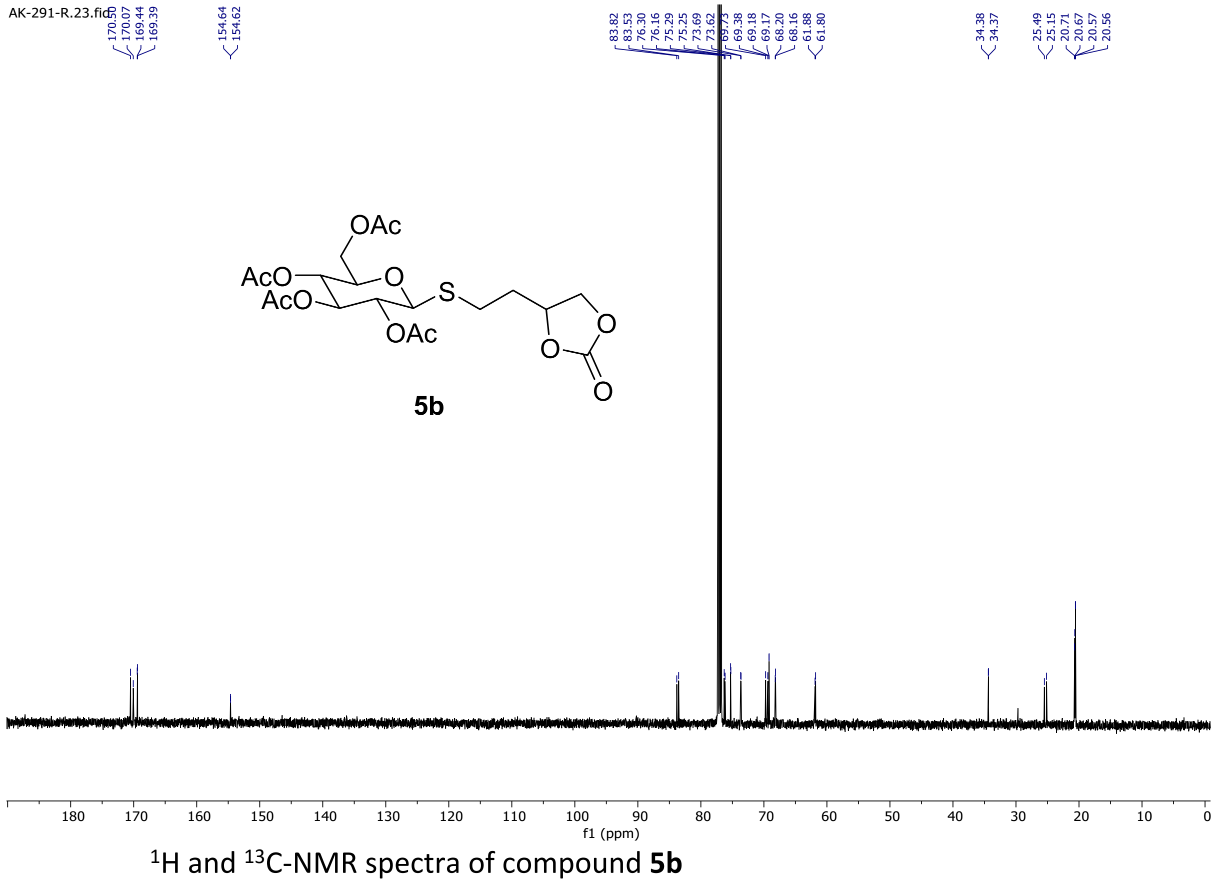

^{1}H and ^{13}C-NMR spectra of compound **5b**

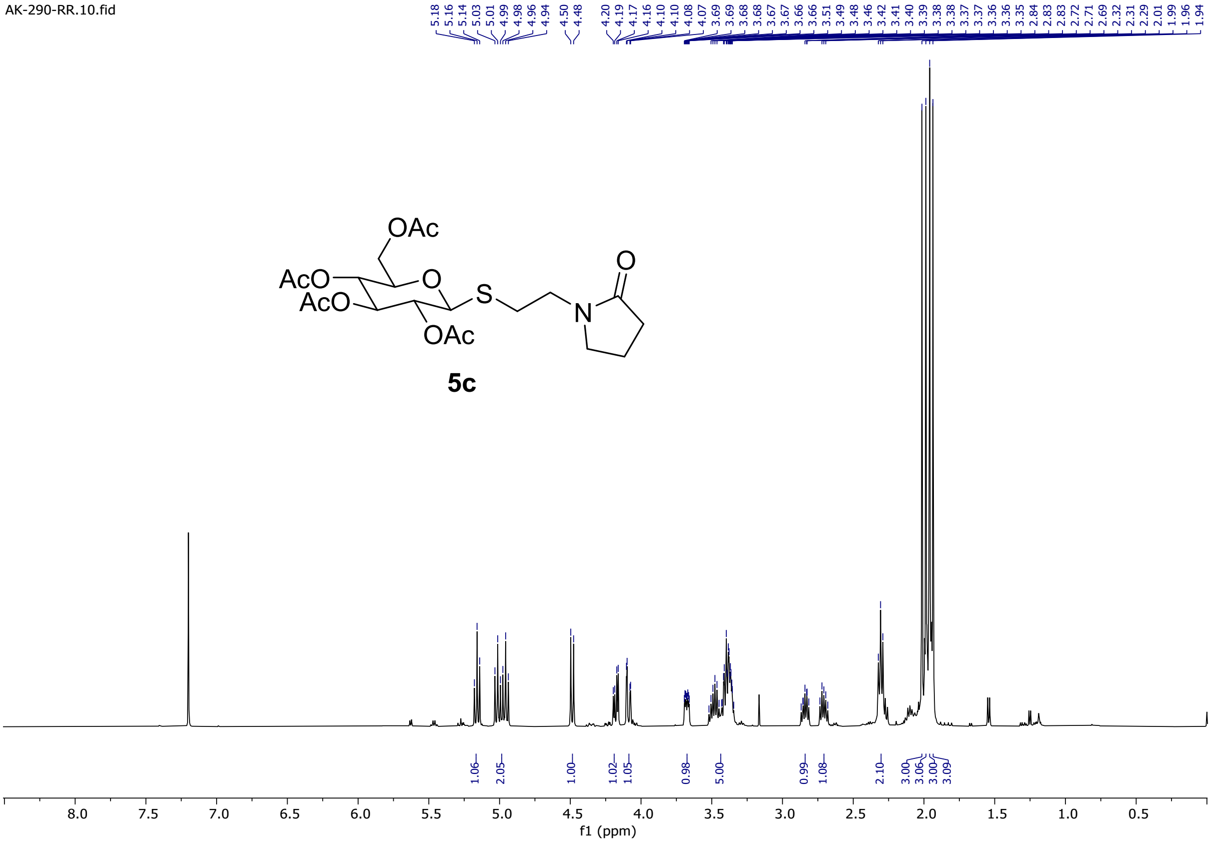

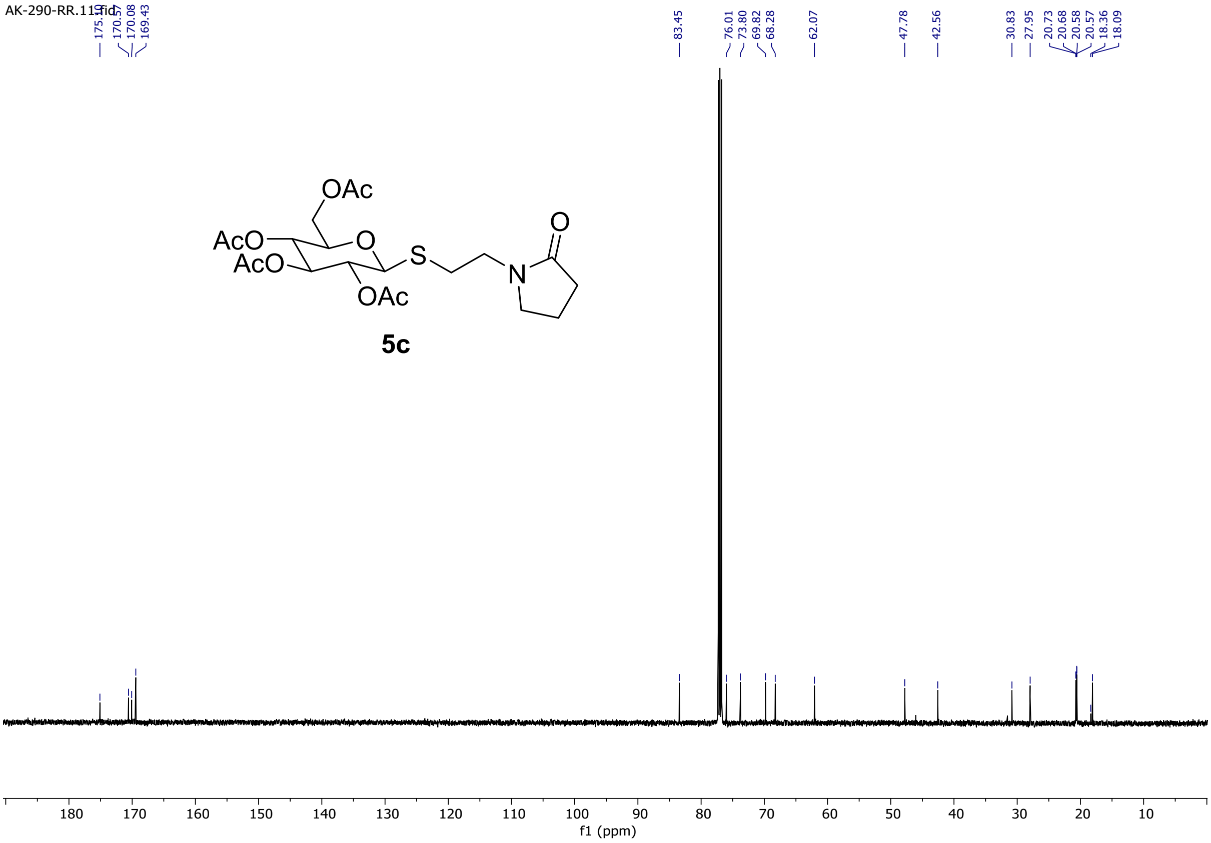

^{1}H and ^{13}C-NMR spectra of compound **5c**

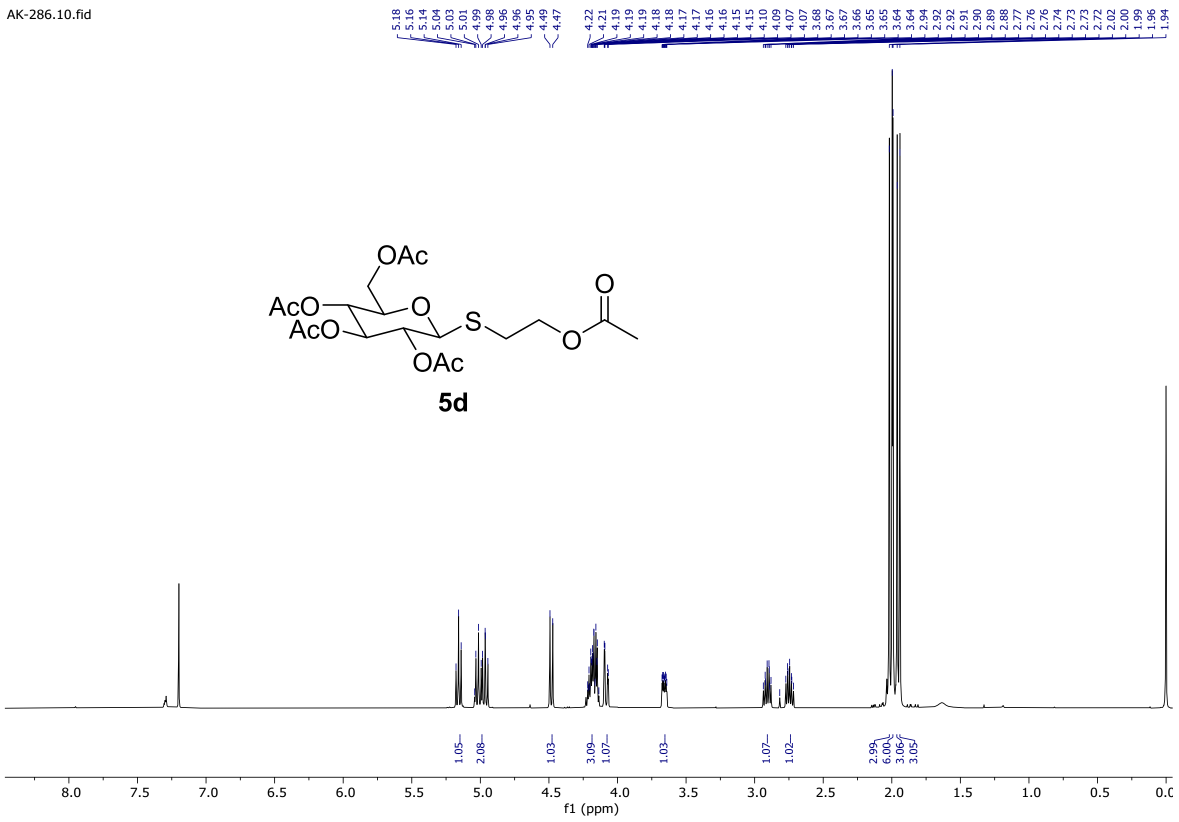

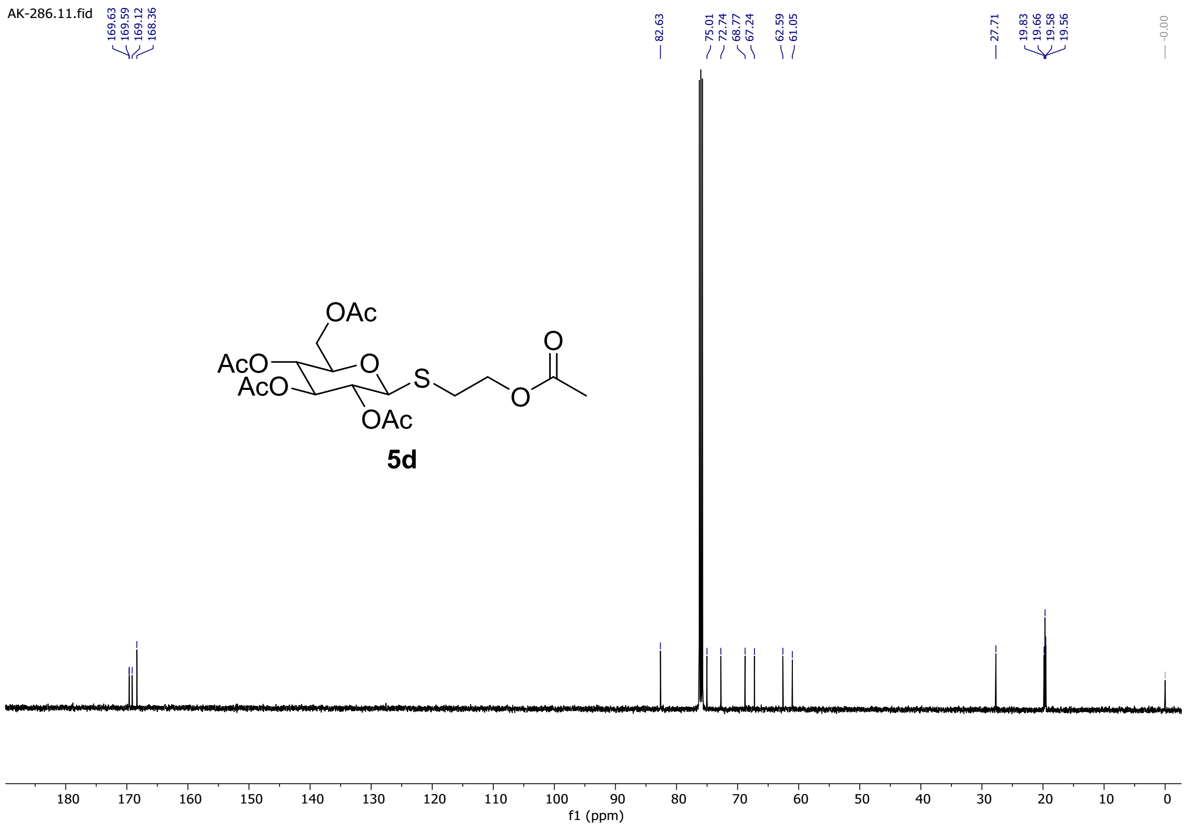

^{1}H and ^{13}C-NMR spectra of compound **5d**

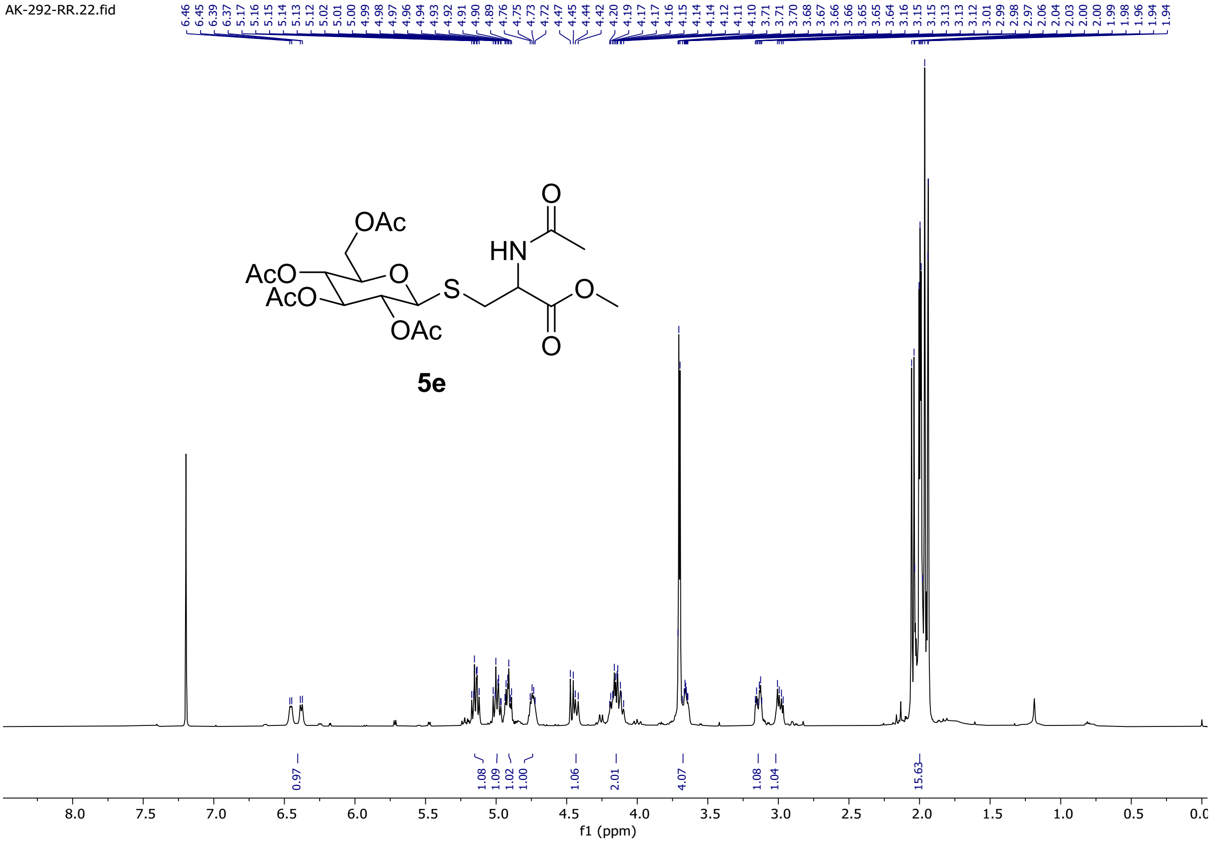
AK-292-RR.22.fid
OAc
AcO
AcO
O
S
OAc
HN
O
O
O
5e
0.97
1.08
1.09
1.02
1.00
1.06
2.01
4.07
1.08
1.04
15.63
f1 (ppm)

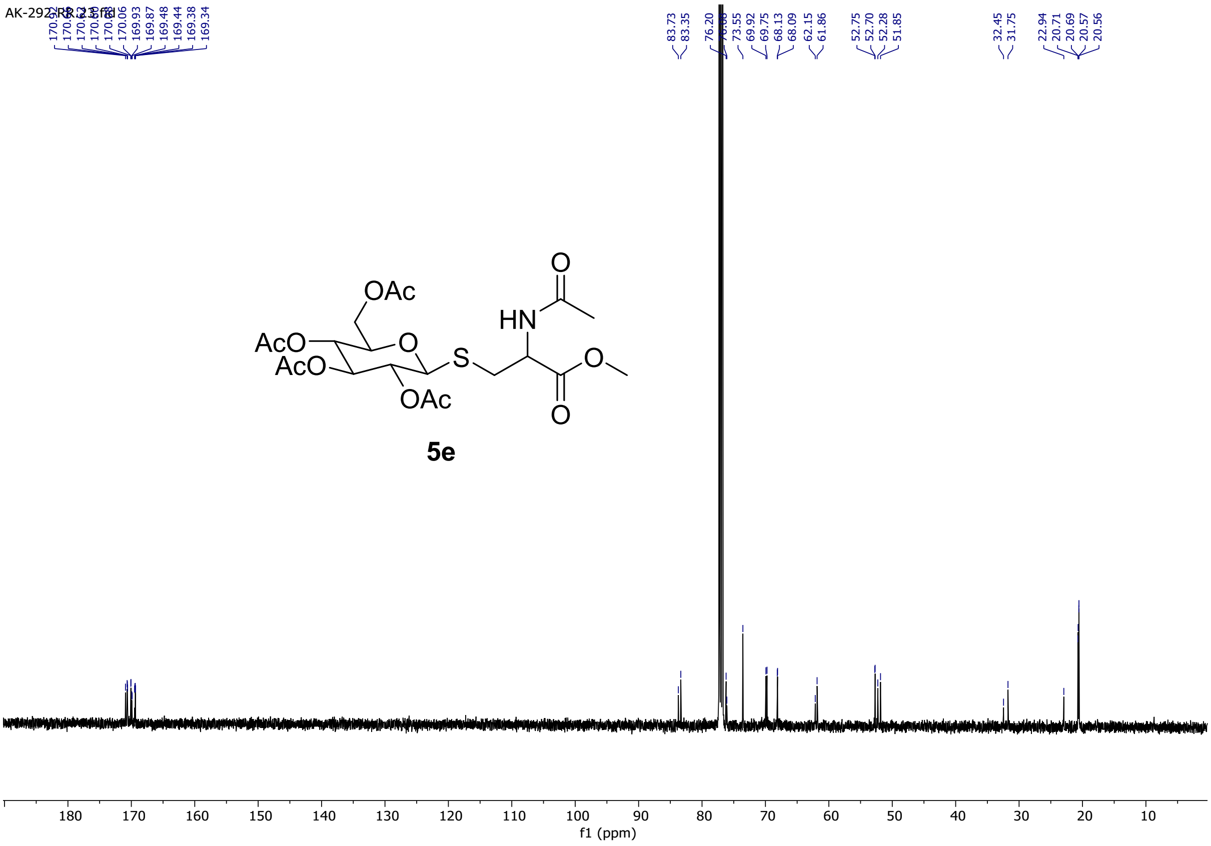
83.73
83.35
76.20
73.55
69.92
69.75
68.13
68.09
62.15
61.86
52.75
52.70
52.28
51.85
32.45
31.75
22.94
20.71
20.69
20.57
20.56
OAc
AcO
AcO
O
S
OAc
HN
O
O
O
5e
f1 (ppm)

^{1}H and ^{13}C-NMR spectra of compound **5e**

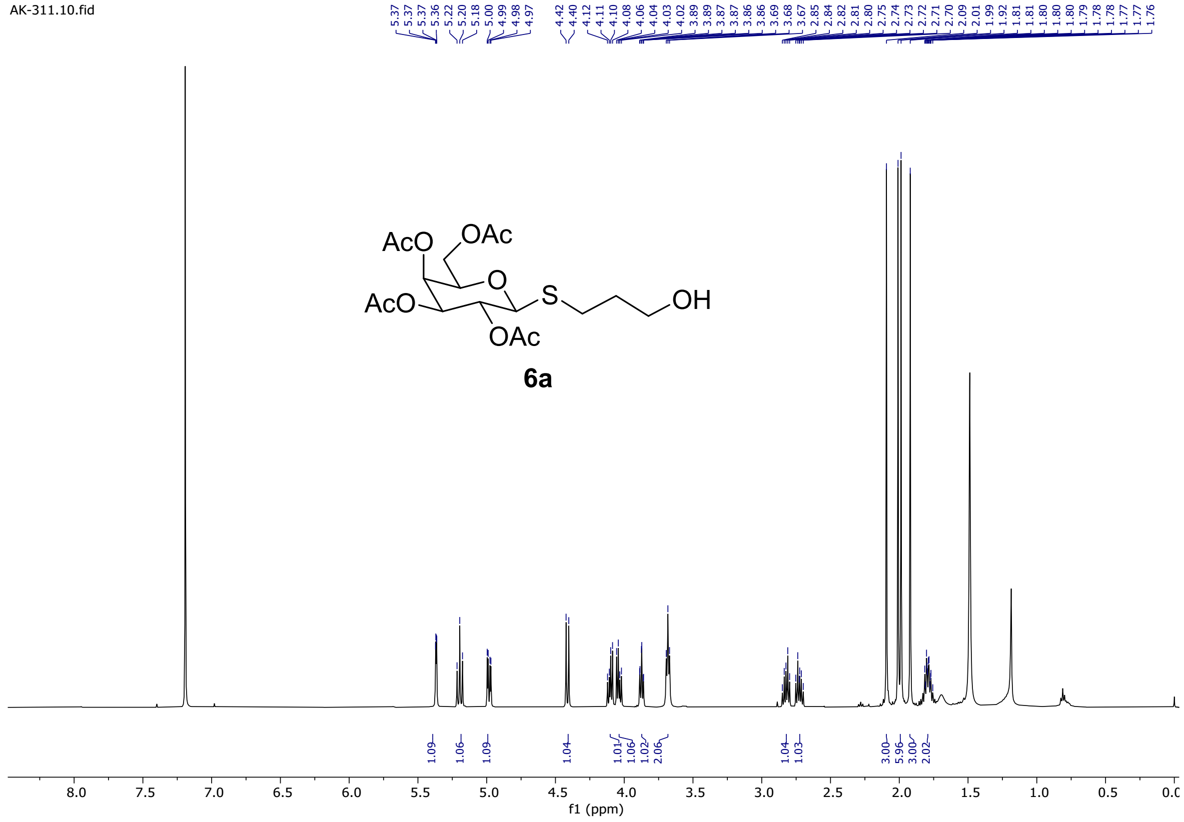

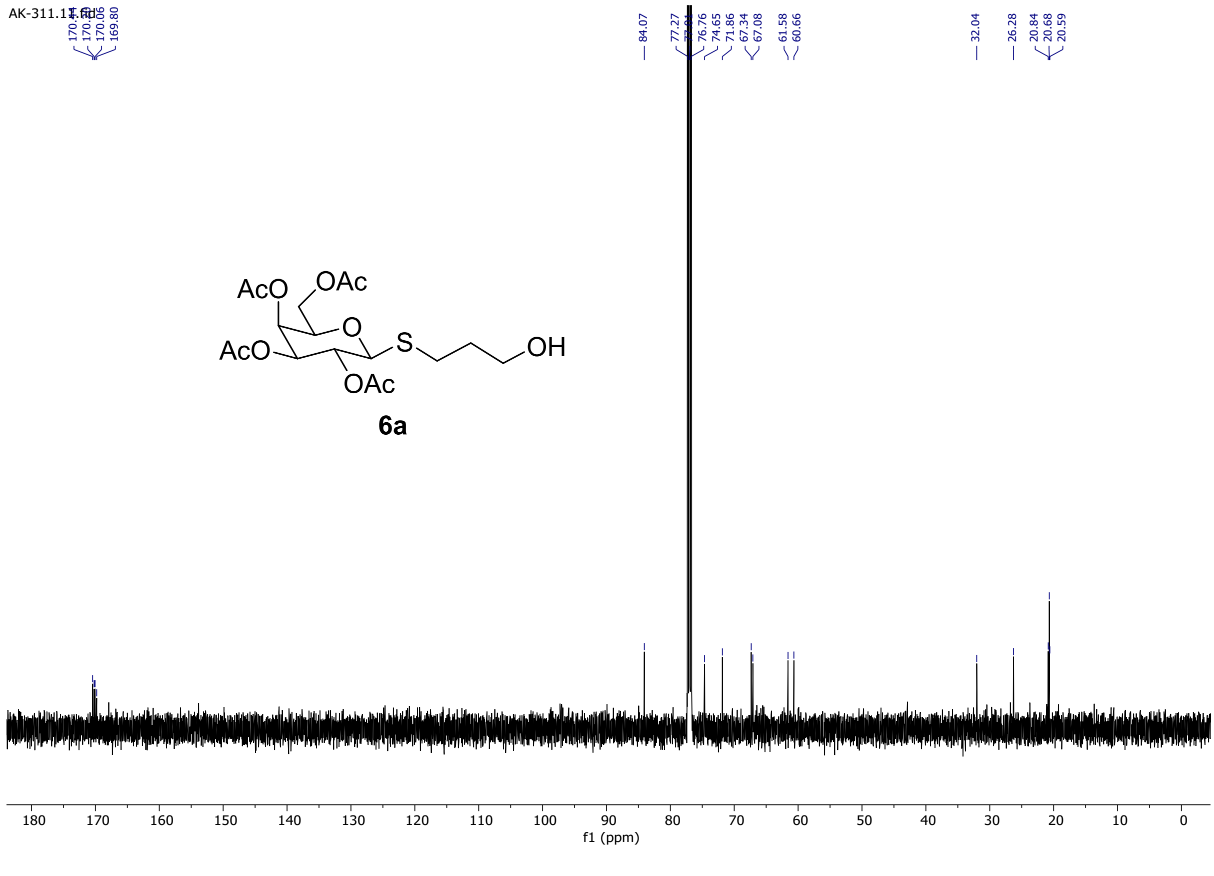

^{1}H and ^{13}C-NMR spectra of compound **6a**

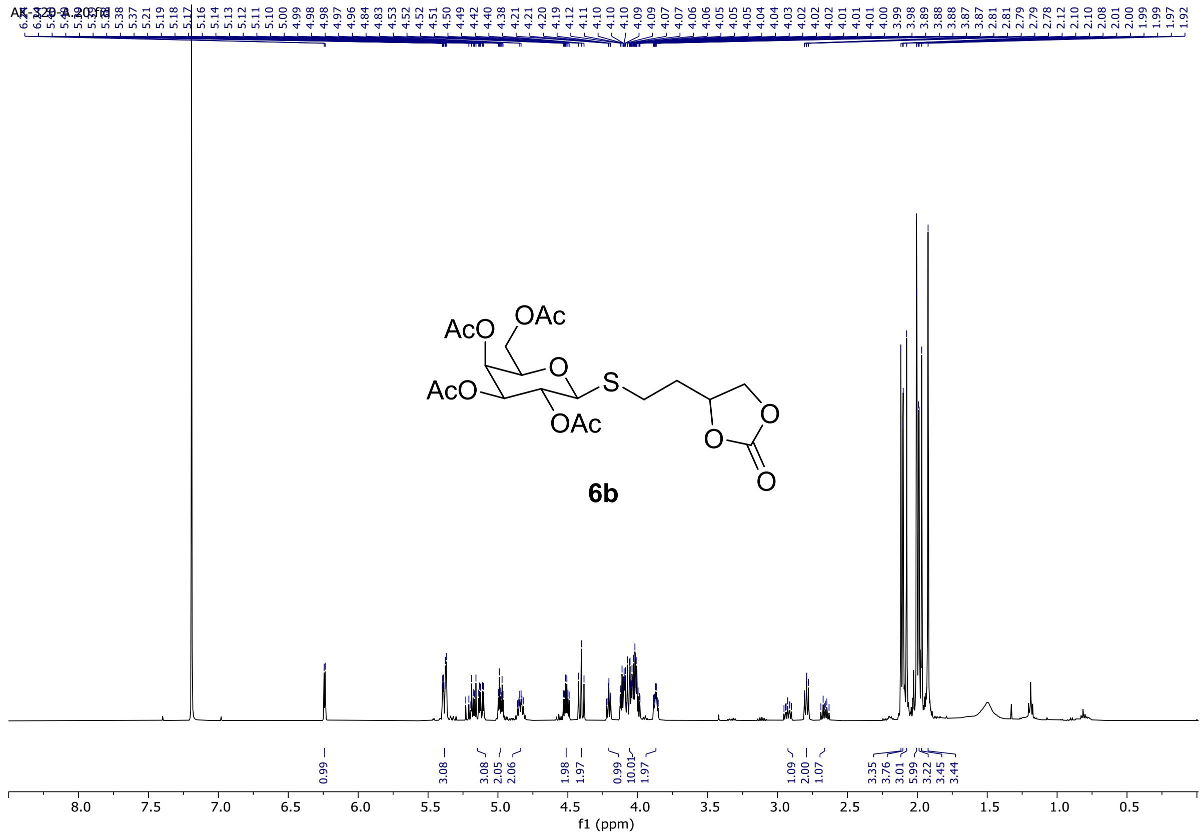

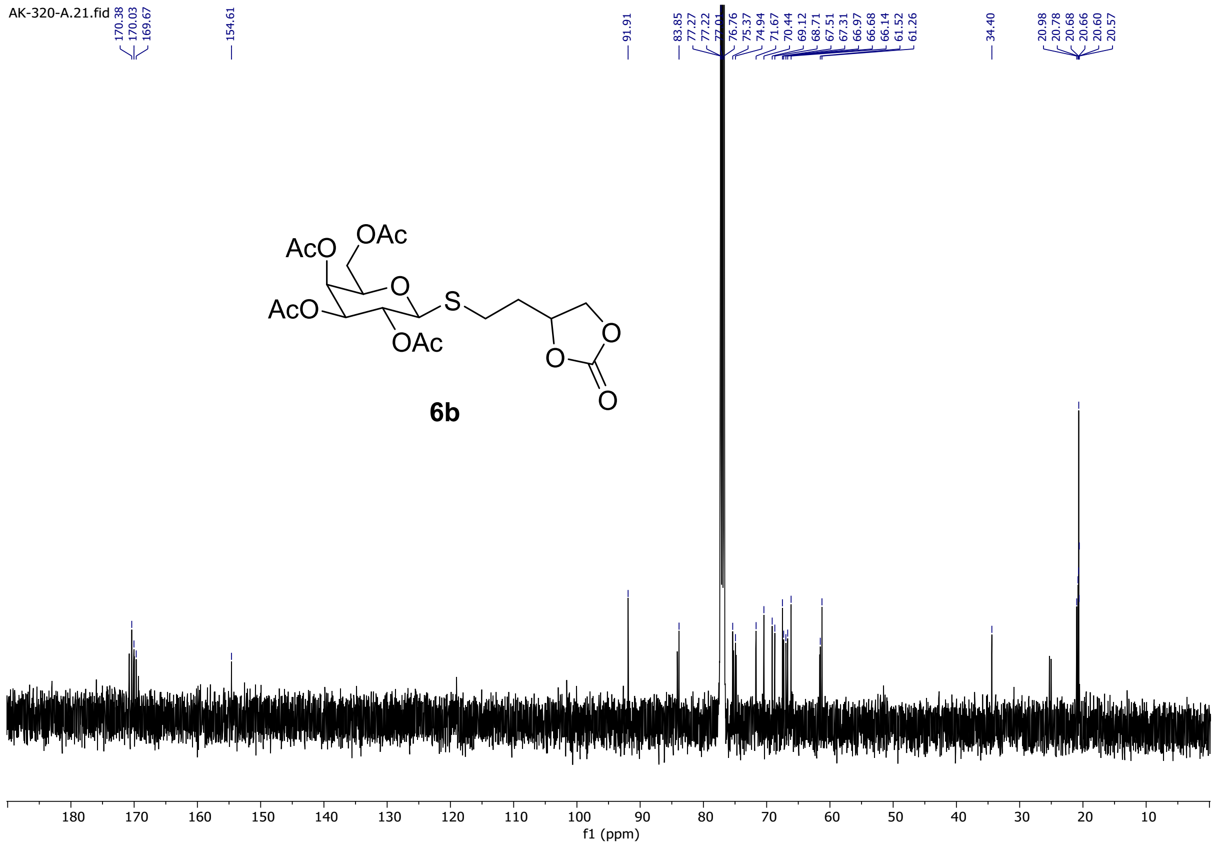

^{1}H and ^{13}C-NMR spectra of compound **6b**

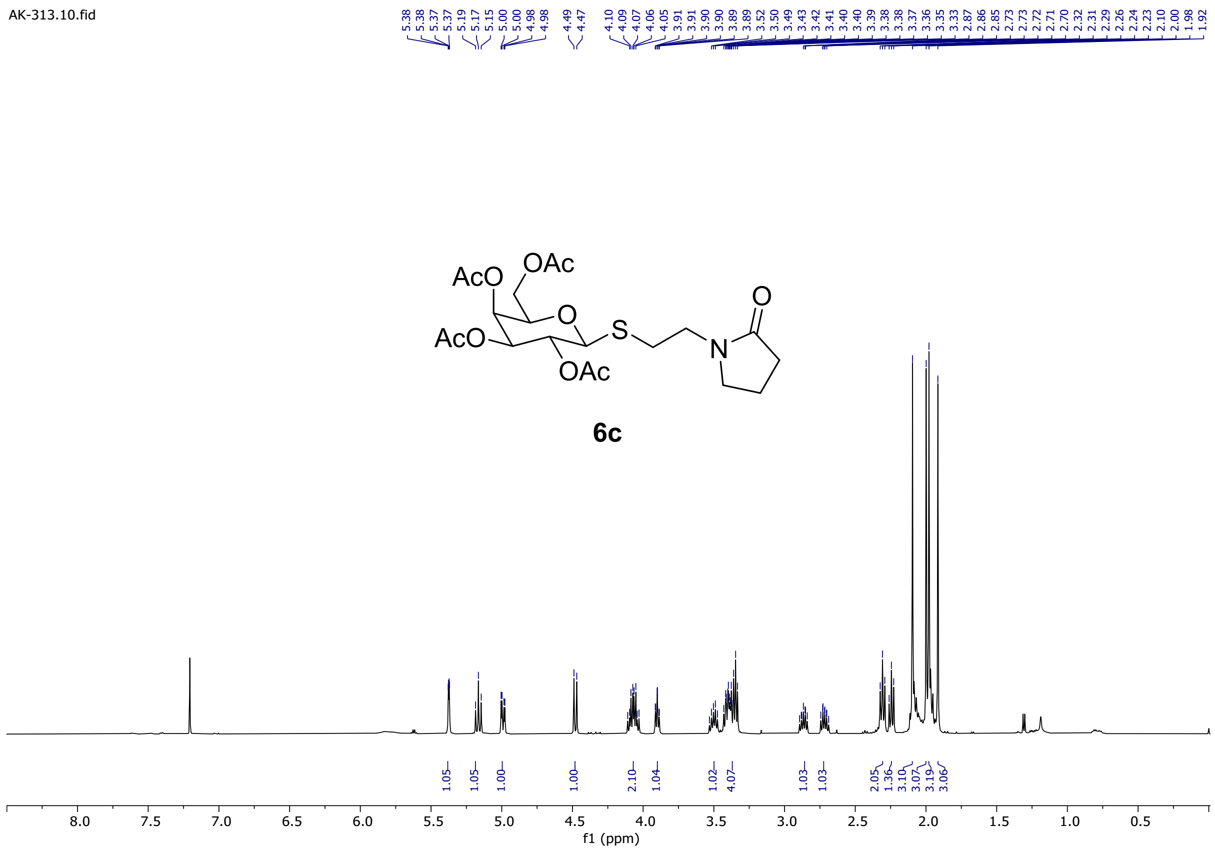
AK-313.10.fid
5.38 5.38 5.37 5.37 5.19 5.17 5.15 5.00 5.00 4.98 4.98 4.49 4.47 4.10 4.09 4.07 4.06 4.05 3.91 3.91 3.90 3.90 3.89 3.89 3.52 3.50 3.49 3.43 3.42 3.41 3.40 3.39 3.38 3.38 3.37 3.36 3.35 3.33 2.87 2.86 2.85 2.73 2.73 2.72 2.71 2.70 2.32 2.31 2.29 2.26 2.24 2.23 2.10 2.00 1.98 1.92
AcO
OAc
AcO
O
S
N
O
OAc
6c
1.05 1.05 1.00 1.00 2.10 1.04 1.02 4.07 1.03 1.03 2.05 1.36 3.10 3.07 3.19 3.06
f1 (ppm)

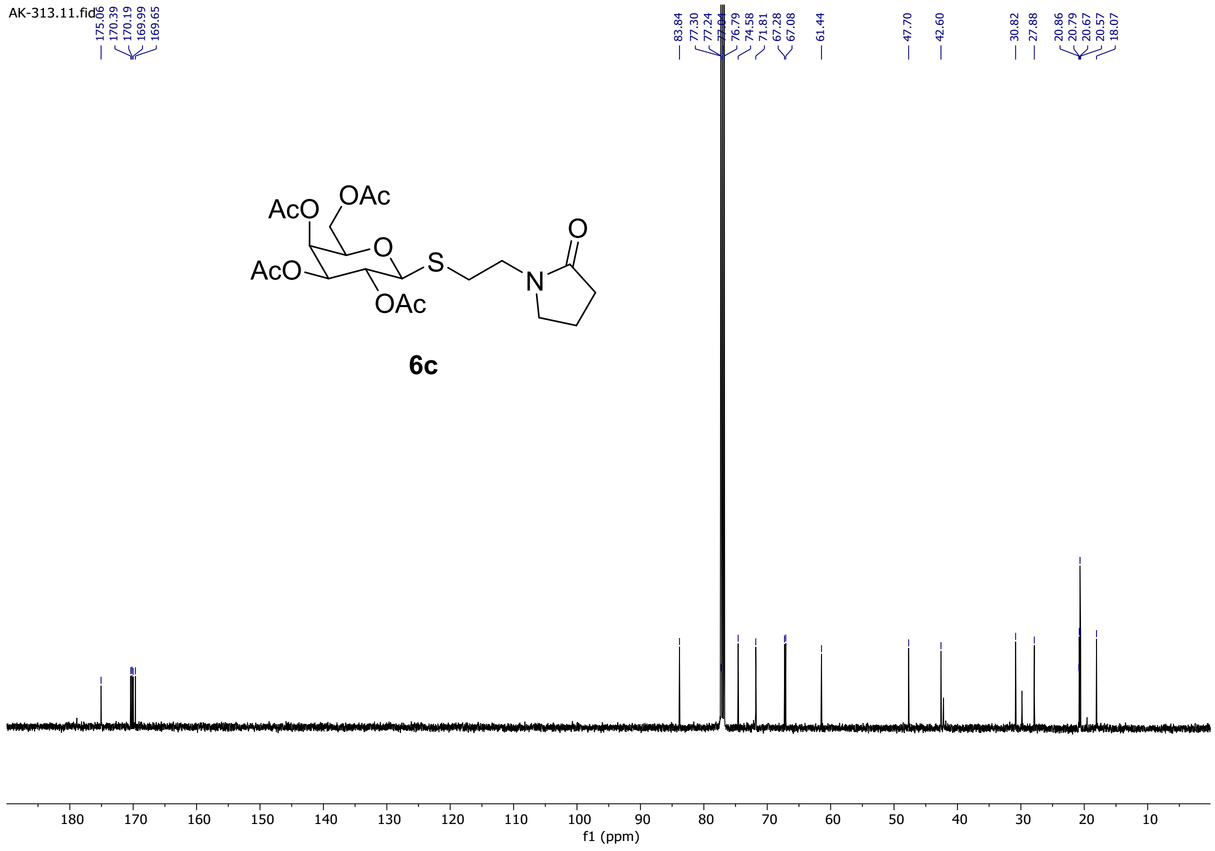
AK-313.11.fid
175.06 170.39 170.19 169.99 169.65
83.84 77.30 77.24 77.04 76.79 74.58 71.81 67.28 67.08 61.44 47.70 42.60 30.82 27.88 20.86 20.79 20.67 20.57 18.07
AcO
OAc
AcO
O
S
N
O
OAc
6c
f1 (ppm)

^{1}H and ^{13}C-NMR spectra of compound **6c**

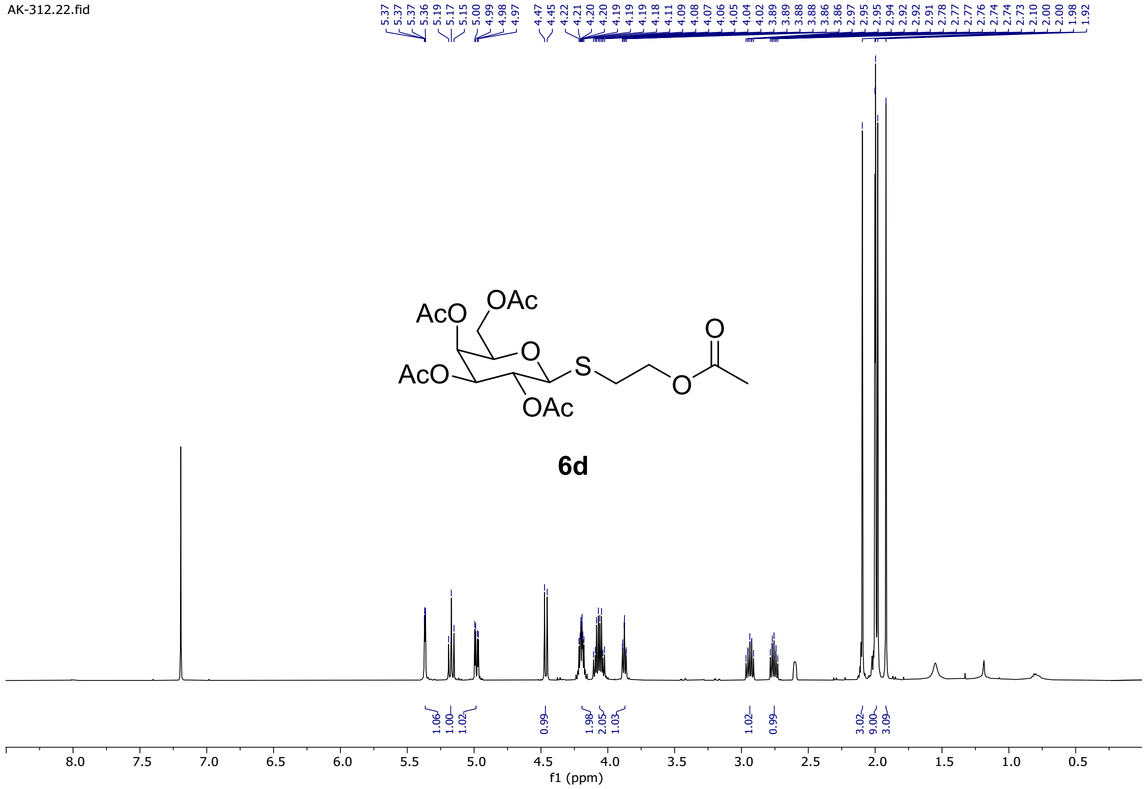

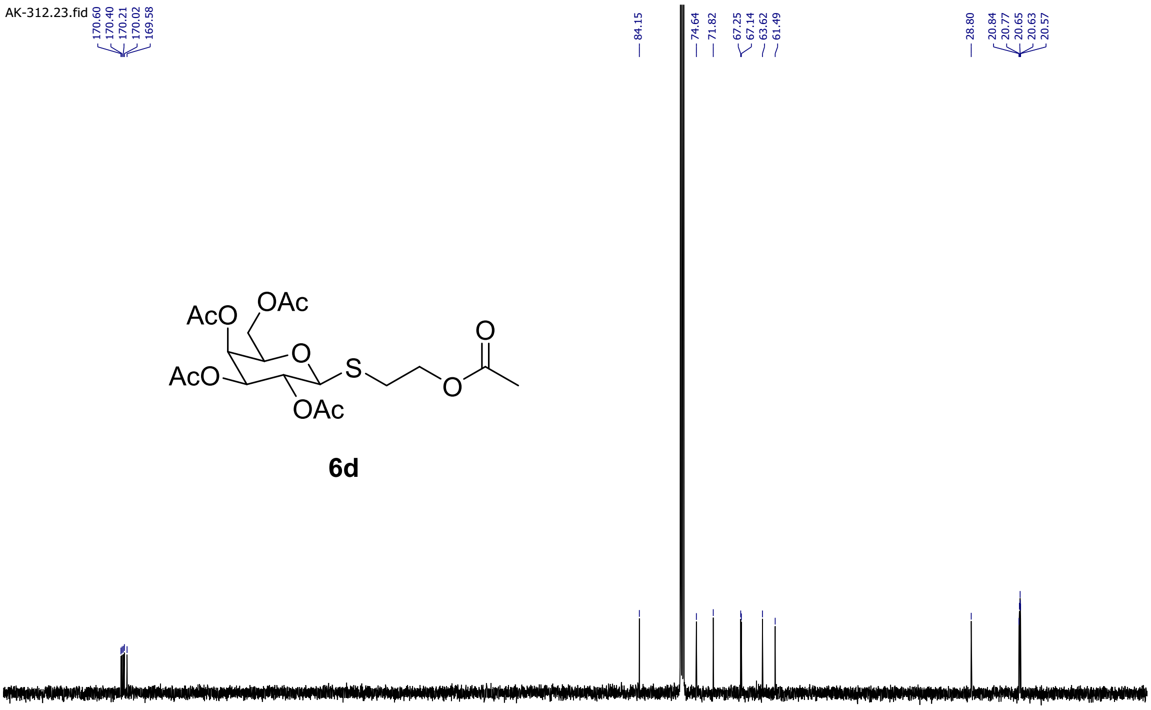

^{1}H and ^{13}C-NMR spectra of compound **6d**

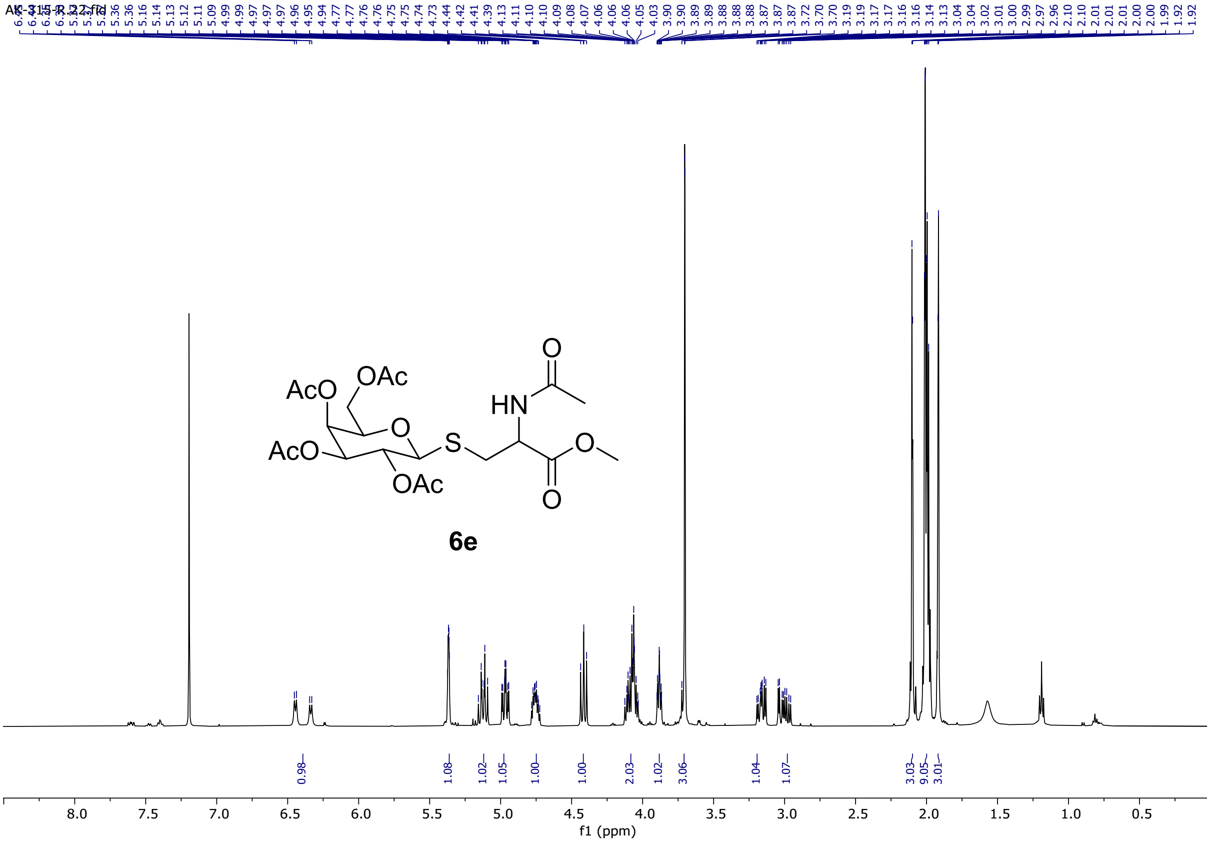
AK-315-R.22.fid
5.36 5.36 5.16 5.14 5.13 5.12 5.11 5.09 4.99 4.99 4.97 4.97 4.96 4.95 4.94 4.77 4.77 4.76 4.76 4.75 4.75 4.74 4.73 4.44 4.42 4.41 4.39 4.13 4.11 4.10 4.10 4.09 4.08 4.07 4.06 4.06 4.06 4.05 4.03 3.90 3.90 3.89 3.89 3.88 3.88 3.88 3.87 3.87 3.72 3.70 3.70 3.19 3.19 3.17 3.17 3.16 3.16 3.14 3.13 3.04 3.04 3.02 3.01 3.01 3.00 2.99 2.97 2.96 2.10 2.10 2.01 2.01 2.00 1.99 1.92 1.92
AcO
OAc
O
HN
O
S
O
AcO
OAc
O
6e
0.98
1.08
1.02
1.05
1.00
1.00
2.03
1.02
3.06
1.04
1.07
3.03
9.05
3.01
8.0 7.5 7.0 6.5 6.0 5.5 5.0 4.5 4.0 3.5 3.0 2.5 2.0 1.5 1.0 0.5
f1 (ppm)

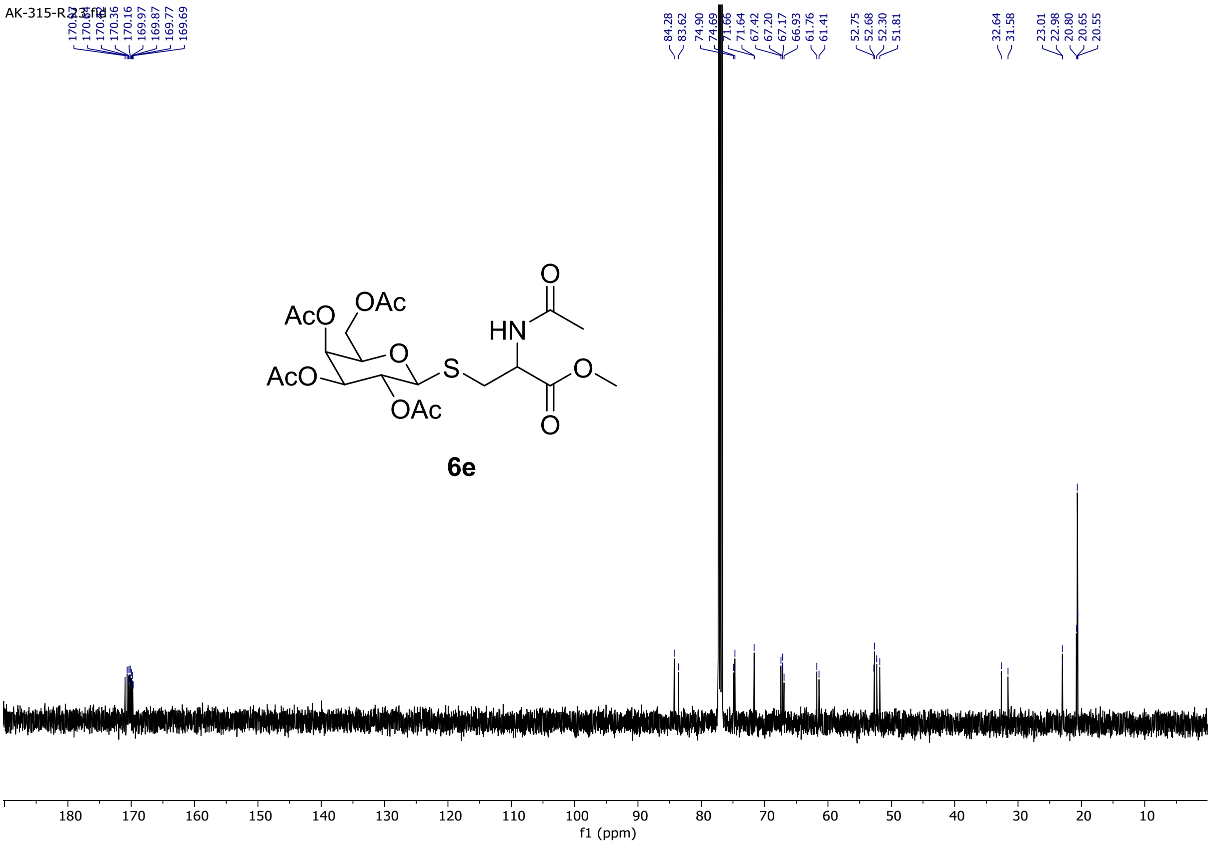
AK-315-R.23.fid
170.16 169.97 169.87 169.77 169.69
84.28 83.62 74.90 74.69 71.64 67.42 67.20 67.17 66.93 61.76 61.41 52.75 52.68 52.30 51.81 32.64 31.58 23.01 22.98 20.80 20.65 20.55
AcO
OAc
O
HN
O
S
O
AcO
OAc
O
6e
180 170 160 150 140 130 120 110 100 90 80 70 60 50 40 30 20 10
f1 (ppm)

^{1}H and ^{13}C-NMR spectra of compound **6e**

Supplementary Reference

1 Feng, G.-J. *et al*. *European Journal of Organic Chemistry* **2021**, 2940-2949 (2021).
